# Metal-Based G-Quadruplex Binders for Cancer Theranostics

**DOI:** 10.3390/ph14070605

**Published:** 2021-06-23

**Authors:** Elisa Palma, Josué Carvalho, Carla Cruz, António Paulo

**Affiliations:** 1 C2TN-Centro de Ciências e Tecnologias Nucleares, Instituto Superior Técnico, Universidade de Lisboa, Estrada Nacional 10, 2695-066 Bobadela LRS, Portugal; elisa@ctn.tecnico.ulisboa.pt; 2 CICS-UBI-Centro de Investigação em Ciências da Saúde, Universidade da Beira Interior, Av. Infante D. Henrique, 6200-506 Covilhã, Portugal; josueocarvalho@gmail.com (J.C.); carlacruz@fcsaude.ubi.pt (C.C.); 3 DECN-Departamento de Engenharia e Ciências Nucleares, Instituto Superior Técnico, Universidade de Lisboa, Estrada Nacional 10, 2695-066 Bobadela LRS, Portugal

**Keywords:** G-quadruplexes, telomerase, oncogene promoters, cancer theranostics, metal complexes, fluorescent probes, radioprobes

## Abstract

The ability of fluorescent small molecules, such as metal complexes, to selectively recognize G-quadruplex (G4) structures has opened a route to develop new probes for the visualization of these DNA structures in cells. The main goal of this review is to update the most recent research efforts towards the development of novel cancer theranostic agents using this type of metal-based probes that specifically recognize G4 structures. This encompassed a comprehensive overview of the most significant progress in the field, namely based on complexes with Cu, Pt, and Ru that are among the most studied metals to obtain this class of molecules. It is also discussed the potential interest of obtaining G4-binders with medical radiometals (e.g., ^99m^Tc, ^111^In, ^64^Cu, ^195m^Pt) suitable for diagnostic and/or therapeutic applications within nuclear medicine modalities, in order to enable their theranostic potential.

## 1. Introduction

### 1.1. G4 Structures: General Aspects and Relevance for Cancer Theranostics

#### 1.1.1. G4 Structures

**Figure 1:**
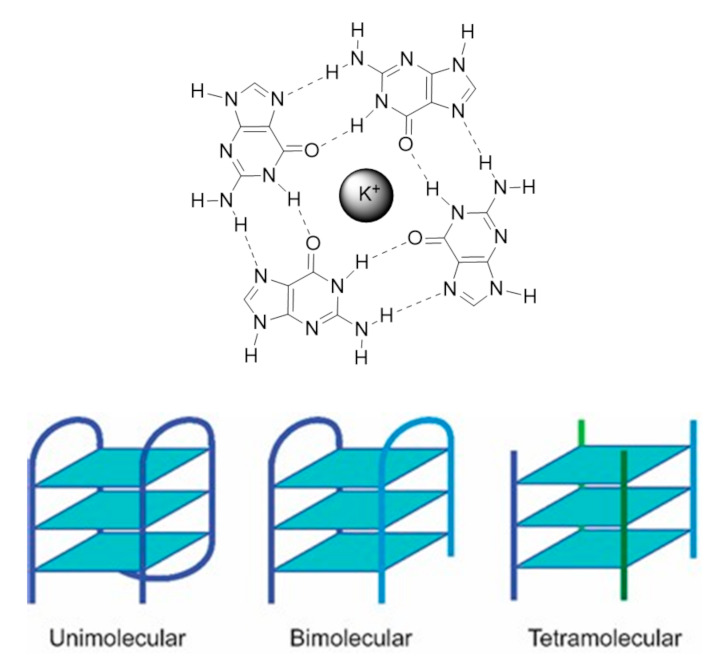
A guanine quartet exhibiting the hydrogen bonding interactions between the Watson-Crick and Hoogsteen faces of the guanine bases, and the metal cation located at the center of the quartet. Below, different G4 arrangements in terms of strand stoichiometry, namely unimolecular, biomolecular, and tetramolecular.

Nucleic acids are highly dynamic biopolymers that have the potential to fold into a variety of structures other than the famous double helix model introduced by James Watson and Francis Crick in 1953 [1]. This expands the array of functions and processes in which nucleic acids are involved, showing that DNA is much more than a ‘simple’ storage of genetic information as once believed. Indeed, only 3% of the human genome is expressed into proteins but the vast majority of the human genome (80%) is involved in the regulation of some kind of biochemical process [2]. One of the functionally important noncanonical nucleic acid secondary structures are G4s. The G4 structure was uncovered almost a decade after the discovery of the double helix model, in 1962 [3], but the first observation of the formation of G4s dates back to 1910 [4]. G4s can be formed under physiologically relevant conditions by guanine-rich DNA or RNA sequences. Its core motif, called the G-tetrad or G-quartet, consists of the square planar arrangement of four guanines held by Hoogsteen hydrogen bonds (Figure 1) [5]. 

The G-quartet is stabilized by monovalent cations such as potassium (K^+^) and sodium (Na^+^) and to a lesser extent lithium (Li^+^), which coordinate with the central electronegative carbonyl O6 atoms of the G-quartet core [5]. G4 structures formed in the presence of K^+^ are considered to be biologically more relevant due to its higher intracellular concentration (≈140 mM) and its *quasi*-perfect fitting in the cavity formed between adjacent G-quartets [6]. G4s display a wide variety of topologies, depending on the sequence, loop size, strand stoichiometry (intramolecular or intermolecular), strand polarity and orientation (parallel or antiparallel), and the cation present in solution [7].

Regarding the strand stoichiometry, G4s can be unimolecular (intramolecular), bimolecular, and tetramolecular (intermolecular), whether these are formed by one, two, or four nucleic acid molecules, respectively. Three strand arrangements are also possible, and their existence has been demonstrated in vitro [8]. As a rule of thumb, G-rich sequences with potential to fold into unimolecular G4s are comprised of four consecutive G-tracts, separated by three loop regions of different lengths and sequences, while bimolecular G4s are formed by sequences with two or three G-tracts [9]. In their turn, tetramolecular structures are formed by single-repeat G-tracts containing sequences [9]. Most biologically relevant G4s are intramolecular structures, with three G-quartets arrangements being the most common. The guanines in each G-quartet can adopt *anti* or *syn* glycosidic orientations, which define the G4 topology [5]. Parallel G4s have all the guanines in *anti*-conformation, being all the strands in the same direction relatively to each other; antiparallel G4s have both *anti* and *syn* guanines, with the strands in opposite directions [10]. There is an additional topology, called hybrid-type, where three strands are in one direction and the fourth in the opposite direction, and the stacked G-quartets adopt *anti-anti-anti-syn* and *syn-syn-syn-anti* alignments (3+1) [10]. Tetramolecular structures are usually parallel G4s while unimolecular G4s exhibit a high degree of conformation diversity with a variety of loop conformations (propeller, diagonal, and edgewise loops) and topologies [10]. The stability and conformation of G4s strongly depend on the cation, as it also contributes to the structural polymorphism. One interesting behavior of G4s is the topology transition observed under different ionic conditions. The same sequence can adopt different G4 conformations with K^+^ and Na^+^, as is the case of telomeric DNA sequences [11]. Interestingly, RNA G4s are generally independent of the cation identity being less diverse in terms of folding topologies as they adopt an exclusively parallel conformation due to the 2’-hydroxyl group of the ribose, which locks the guanines in *anti*-position [12].

The availability of the complete human genome provided the needed data for a systematic search of G4-prone guanine-rich sequences employing algorithms using sequence motifs such as G_≥3_N_x_G_≥3_N_x_G_≥3_N_x_G_≥3_, where N can be any nucleotide within the loop of “x” size [13,14]. A variety of computational analysis carried by different groups found more than 375,000 potential G4 sequences in the human genome. The question if all these sequences can form G4 structures in vivo is yet to be answered. Nonetheless, a critical aspect came from these studies: the G4-prone sequences are widely dispersed in the human genome following a non-random distribution. These are particularly prevalent in the telomeres, the promoter regions of oncogenes, and 5’ untranslated regions [14]. These guanine-rich sequences are located in regions that influence gene metabolism processes, as well as key biological processes such as DNA replication and recombination, gene transcription and translation. This prompted investigations into possible roles of G4s in diseases such as cancer and intensified the research and development of G4-targeted small-molecule ligands as potential therapeutic agents. 

#### 1.1.2. G4 Targets in Cancer Cells

##### G4-Mediated Targeting of Telomerase and Telomere Maintenance

**Figure 2:**
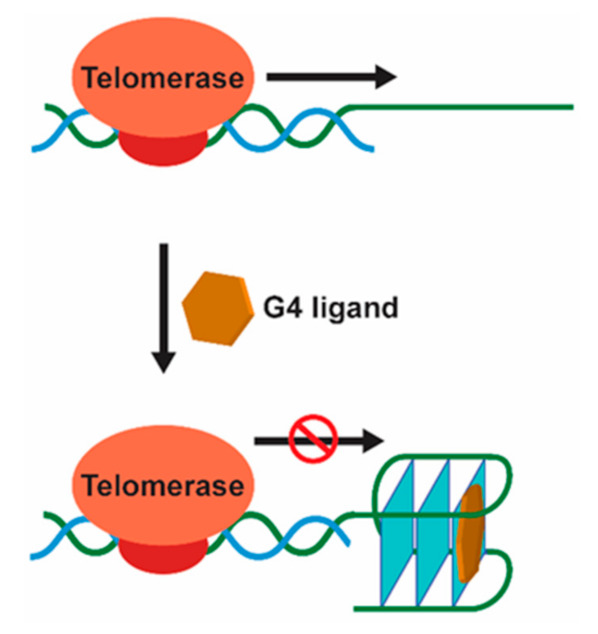
Schematic illustration of indirect telomerase inhibition by G4 ligand at telomeres.

Human telomeric DNA is typically 5–10 kb long duplex DNA consisting of tandem repeats of the motif d[TTAGGG] with a single-stranded 3’ overhang of 30–600 bases [15]. These G-rich single-stranded overhangs were already shown to form discrete G4 arrangements, being the first observation of biologically relevant G4 formation [16]. The length of the duplex portion decreases progressively after each cell division cycle in somatic cells because of the end-replication effect. On the other hand, the single-stranded 3’ overhangs can be elongated by telomerase, an enzyme with reverse transcriptase activity, which is expressed in the majority of cancer cells (80–85%) and primary tumors, thus, maintaining the telomere-length homeostasis [17]. In cancer cells, where telomerase is overexpressed, it maintains the telomere length acting as a tumor promoter and helping the cells to bypass apoptosis and achieve cellular immortality [17]. Thus, the development of small molecules that bind and stabilize telomeric G4 structures, inhibiting the activity of telomerase, is a viable and promising anticancer therapeutic strategy (Figure 2) [18]. 

The human telomeric G4 structure is highly polymorphic and to date several structure models were proposed [19]. The first model was proposed in 1992 for the single-repeat d[TTAGGGT] human telomere sequence, which formed a parallel-stranded G4 in K^+^ solution [20]. Nuclear magnetic resonance (NMR) studies, involving the three-repeat human telomere sequence 5’-GGGTTAGGGTTAGGGT-3’, demonstrated the formation of a hybrid-type 3+1 G4 [21]. This structure formed in the T-loop region where the single-stranded overhang pairs with a complementary portion of the telomeric double-stranded region. Such results supported the biological implications of the G4 formation in the telomeres, and the potential of using small molecules for its stabilization [21]. This concept was first validated through the demonstration that the compound 2,6-diamidodianthraquinone could inhibit the activity of telomerase by interacting with and stabilizing G4 structures [22]. Other compounds followed and showed that G4-induced telomerase inhibition leads to telomere-induced senescence, growth inhibition, and apoptosis [18].

##### Promoter G4 Targeting

**Figure 3:**
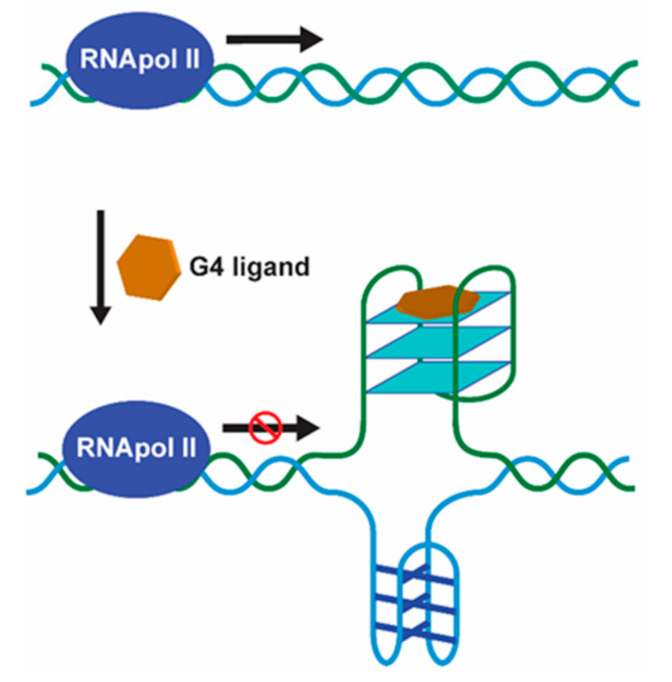
Schematic illustration of transcription regulation of oncogenes by promoter G4 formation mediated by G4 ligand.

The first evidence of the formation of unusual DNA conformations in gene promoters was found in a nuclease hypersensitive element (NHE) within the promoter of *c-myc* [23], which became the paradigm for many subsequent studies. *c-myc* overexpression leads to increased cellular proliferation and differentiation inhibition, and is associated with a variety of human cancers, such as colon, breast, lung, osteosarcoma, glioblastoma, and myeloid leukemia [24]. The NHE region, located upstream of its P1 promoter, is a guanine-rich strand that controls 80–90% of *c-myc* transcription and contains a 27 bp sequence with propensity to fold into a variety of G4 structures [25,26]. The first evidence that G4 structures could act as transcriptional repressor elements came from the use of the cationic porphyrin TMPyP4, which inhibited *c-myc* transcription by stabilizing the G4 structure in its promoter (Figure 3) [27]. 

Since then, several other G4-forming sequences were found in the promoter regions of other oncogenes such as *c-Fos, c-kit,* Kirsten rat sarcoma viral oncogene homolog (*KRAS*)*,* vascular endothelial growth factor (*VEGF*)*,* platelet-derived growth factor α polypeptide (*PDGF-A*)*,* retinoblastoma (*Rb*)*, RET,* hypoxia-inducible factor 1α (*Hif-1α*)*,* B cell lymphoma 2 (*bcl2*)*,* and human telomerase reverse transcriptase (*hTERT*) [28]. These proto-oncogenes are involved in growth and proliferation processes and their proximal promoter regions contain several G and C-rich regions. The formation of G4 structures in these regions is believed to modulate the genes’ transcriptional activity. Moreover, these genes are important in cell signaling and differentiation, metabolic regulation and cancer progression [28]. As a result, the study of promoter G4 structures and their therapeutic implications has raised a lot of attention in the last decade, and several ligands were developed with the ability to regulate gene expression at G4 level [28]. In contrast to the telomeric G4s, promoter G4 structures are mostly of parallel topology and possess unique capping and loop structures [29].

##### RNA G4 Targeting

The formation of G4 structures is also common on RNA nucleic acids. RNA G4 structures are more thermodynamically stable, compact, and less hydrated than their DNA counterparts [12]. The presence of a 2’ hydroxyl group in the ribose moiety produces additional intramolecular interactions within the structure leading to an enhanced stability. The first observation of a human RNA structure was made in 2008 when Xu and collaborators demonstrated that a transcript of telomeric DNA (TERRA) composed of tandem repeats of the motif UUAGGG was able to form stable RNA G4 structures [30]. RNA G4s are also present in noncoding mRNA, as well as in other premature and mature noncoding RNAs such as miRNAs. Following these findings, numerous studies have shown the contribution of RNA G4 to various biological processes, including transcription and translation regulation, mRNA processing, mRNA localization, as well as alternative splicing [12,31]. Due to their broad distribution and function, RNA G4s rapidly emerged as an interesting target for novel anticancer drug development strategies, namely small molecule ligands. The ligands exert their effect by stabilizing or destabilizing RNA G4 structures, which results in the inhibition of translation, restoration of dysregulated levels of RNA molecules in pathology and interference with miRNAs maturation. The unique chemical properties of RNA G4s suggest that it is possible to develop RNA-specific ligands. A recent example is carboxypyridostatin, a pyridostatin (PDS) derivative that exhibits high molecular specificity for RNA G4s over DNA G4s [32]. Selective intervention targeting specifically RNA G4s may be an important therapeutic approach for specific diseases such as ALS/FTD and Fragile X syndrome. 

#### 1.1.3. Control of G4 Formation and Unwinding

**Figure 4:**
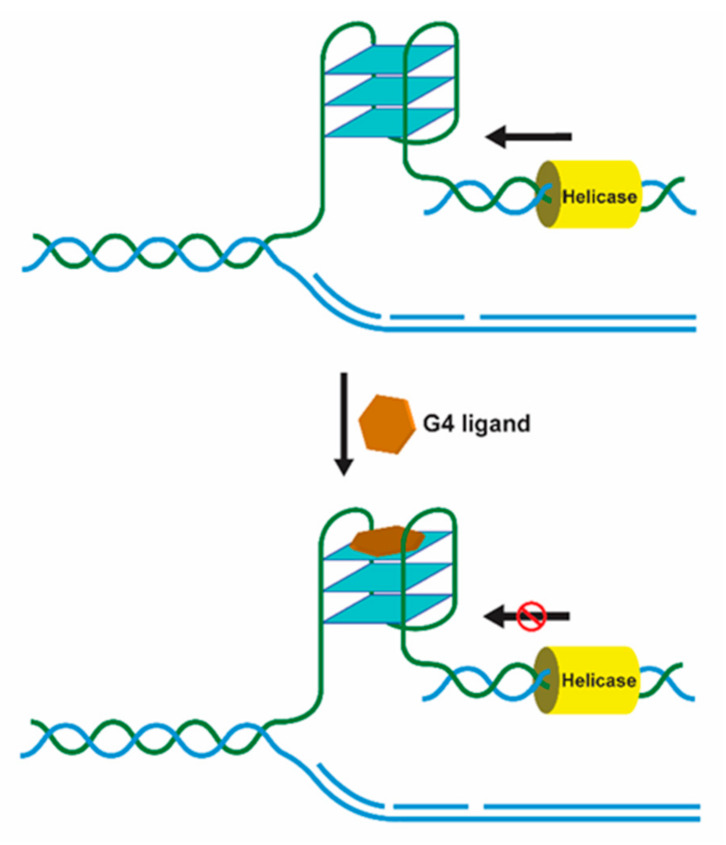
Schematic representation of G4 ligand-dependent modulation of helicase unwinding activity, which in turn affects DNA replication.

Helicases are molecular motors that resolve nucleic acid structures and play important roles in diseases such as cancer and ageing. Helicases are involved in a variety of aspects of cell metabolism such as DNA replication, repair, recombination, transcription, and telomere maintenance [33]. Regardless of the location and function of G4s, timely and controlled formation/unwinding are critical since misregulated G4 structures are detrimental for any given biological process, acting as kinetic traps causing genomic instability [34]. G4 formation is transiently regulated by specific G4-interacting proteins with helicase activity such as WRN, BLM, DHX36, FANCJ, and ScPif [34]. G4 helicases operate during telomere maintenance, DNA replication, and gene transcription. In the absence of specialized helicases such as RecQ helicases, the accumulation of G4 structures in the telomeres is observed, resulting in defects during lagging-strand replication and end-to-end fusions of telomeres [35]. During replication fork progression and DNA replication initiation, G4 helicases are of great importance to efficiently unwind G4 structures that pose obstacles to these processes resulting in genomic and epigenetic instability, if not properly regulated [36]. Pif1, for instance, binds and unwinds G4 structures at the end of S-phase located on the leading strand, suppressing genome instability [37]. During transcription, helicases resolve G4 structures that hinder the activity of RNA polymerases. For instance, helicases XPD and XPB, which bind and unwind G4 structures in vitro, were shown to bind particularly to G4 structures at promoters of highly transcribed gene by a genome-wide ChIP-seq analyses [38]. The helicase activity may be modulated in the presence of G4 ligands being also an interesting target for drug development (Figure 4).

#### 1.1.4. Helicases and Other G4-Binding Proteins

The existence of natural proteins that recognize G4s in vivo, provided important insights in the location of G4 structures in the genome and showed the biological relevance of guanine-rich sequences [39]. These proteins bind, promote, or disrupt the G4 structure formation, and over 30 proteins have been reported so far with the ability to bind G4 structures in telomeric regions, promoter regions, and RNA G4s [40]. 

POT1 (protection of telomeres 1) binding to DNA disrupts G4 formation at the telomeric G-rich overhangs, promoting telomerase activity [39]. Replication protein A (RPA), which is involved in replication, repair, and recombination, also demonstrated unfolding activity of G4 structures, competing with POT1 [41]. Other proteins are involved in transcriptional regulation such as poly [ADP-ribose] polymerase 1 (PARP-1), which was found to regulate *c-kit* and *KRAS* transcription [42]. PARP-1 gets catalytically activated upon interacting with the parallel G4 structures within the oncogene promoters. Nucleolin (NCL) is a cellular protein reported for its G4-dependent functions, such as induction of neurodegenerative diseases, tumor and virus processes activation and regulation [43]. NCL is considered a molecular chaperone that regulates G4 folding and was shown to bind *c-myc* G4, promoting its stability and inhibiting its promote-driven transcription [44]. Other G4-binding proteins that were shown to also interact with RNA G4 structures are hnRNP, serin/arginine-rich splicing factor (SRSF) 1 and 9, splicing factor U2AF, ribosomal proteins, and the RNA helicase associated with AU-rich element (RHAU) [40]. These proteins act at RNA level by alleviating the G4-induced translational block and modulating alternative splicing.

#### 1.1.5. The Therapeutic Relevance of G4s

G4s are associated with mechanisms that control telomere biology, transcriptional regulation of cancer-related genes, replication, and genome instability [7]. Furthermore, DNA G4s, RNA G4s, and i-motifs are present in cancer-promoting genes interacting with transcription factors or impairing polymerase progression along its template; also, experimental data has shown a higher presence of G4 structures in cancer states compared with normal states, which may favor G4s as molecular targets for cancer [45]. For example, antibody staining of human stomach and liver cancer tissues demonstrated higher presence of DNA G4 structures compared to the corresponding non-neoplastic tissues [45]. Moreover, G4 ChIP-seq detected more G4 sites in cancer genes, such as *c-myc, PTEN,* and *KRAS*, of immortalized HaCaT cells but not in their normal human keratinocyte (NHEKs) [46]. The G4 DNA formation is highly dependent on chromatin structure and is frequently found in regulatory, nucleosome-depleted regions in proximity to the transcription start sites of genes that undergo elevated transcription [46]. In addition, RNA G4s differentially influence translation and mediate splicing-associated binding proteins, regulating alternative splicing of numerous important genes in carcinogenesis [47]. The distinct topologies of the distinct G4s enable structure-selective recognition by small ligands. Several G4 ligands have been evaluated for their therapeutic potential as a novel anticancer strategy and have shown antitumoral activity in vitro and through xenograft models [47]. G4 ligands such as pyridostatin and RHPS4 promotes growth arrest in human cancer cells by inducing replication- and transcription-dependent DNA damage [48]. Moreover, G4 ligands have also shown synergy with DNA damaging therapies in ATR-X-deficient glioma cell models [49].

Some G4 ligands are even in clinical trials. Of particular note is CX-5461, which has recently entered clinical trials for breast cancer patients with BRCA-deficient tumors [50]. CX-3543, also named quarfloxin, passed Phase II trials as a candidate therapeutic agent against several tumors, but Phase III trials were not completed due to its high binding to albumin [51]. However, it is normal to assume that most of these G4 ligands will regulate expression of many genes because they will bind other G4s. Based on that, transcriptional profiling of the cancer cells/tissues treated with G4 ligand must be done to identify the affected pathways and suggest cancers best suited for efficacy studies. 

### 1.2. Metal Complexes in the Design of G4 Binders

Metal complexes have a very broad range of structural and electronic properties that can be exploited to design best performing G4 binders, namely in terms of binding affinity and selectivity. In particular, the metal center can play an important role in the structural organization of the ligands in specific geometries and relative orientations for optimal G4 binding. For this optimization, different coordination geometries can be considered by changing the metal center and ligands/chelators. In addition, the electron withdrawing properties of metals also afford electron-poor systems, which can generate stronger π interactions with G-quartets. Moreover, the electropositive metal can, in principle, be positioned at the center of a G-quartet, thereby increasing the electrostatic stabilization by substituting the alkali metal cation that would normally occupy this site. Altogether, these features make metal complexes advantageous when compared with their organic counterparts since the geometric arrangement and cationic charge localization can be determinant to establish a specific G4 binding.

**Figure 5:**
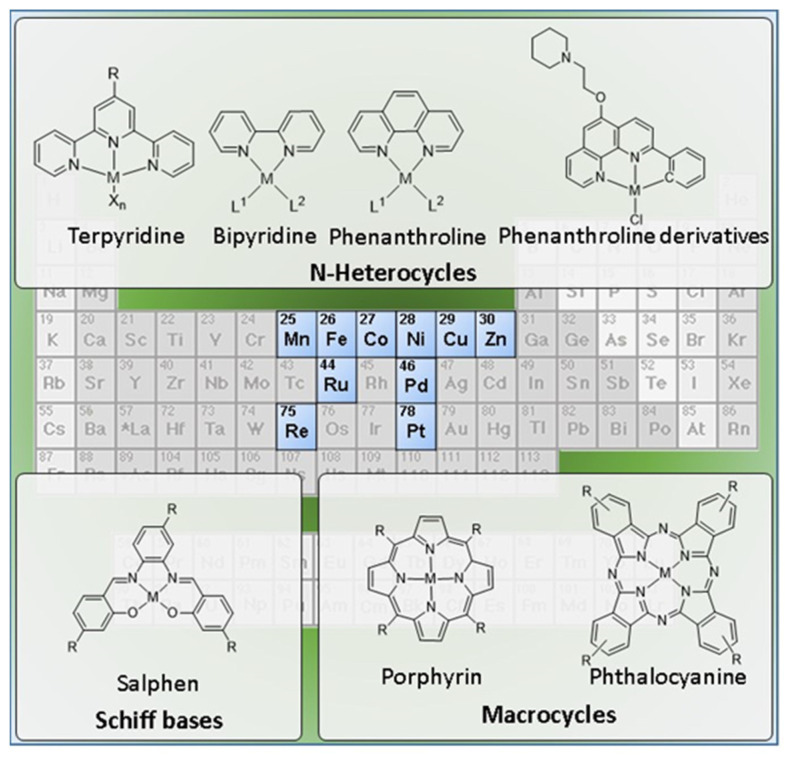
Periodic table highlighting the metals used for the design of complexes with G4-DNA binding affinity, as described in this review, and representative examples of most common structures.

These advantages gave rise to a considerable interest on metallated G4 binders, as recently reviewed elsewhere [52,53,54,55,56,57,58]. In these studies, the complexes of divalent metals (e.g., Zn(II), Cu(II) or Pt(II)) capable of forming square-planar complexes have assumed a prominent importance. Nevertheless, many other d-transition metals, and to a less extent also f-transition metals, have been used in the design of G4 binders (Figure 5).

This research work led to the introduction of diversified libraries of metal complexes, and the study of their interaction with the target DNA contributed to establish structure–activity relationships useful for a more rational design of G4 DNA binders. Most of the studies have focused on planar molecules containing a π-delocalized system, which have the ability to interact through π stacking with G-quartets. It was also shown that the interaction of this class of complexes with the grooves and loops of DNA (containing negatively charged backbone phosphates) may also be enhanced due to the presence of pendant substituents bearing a positive charge [59,60,61]. This strategy is also valid for most organic molecules tested so far, but metal complexes have the ability to interact with G4 DNA through additional modes, such as direct coordination to bases or the phosphate backbone. 

Fluorescent metal complexes have been applied in multiple areas of science and technology, including as sensors in different (bio)analytical applications and as cancer imaging probes [62,63,64,65,66,67,68,69,70]. In this context, the fluorescent properties of some metal-based complexes that specifically recognize G4 structures make them useful also as probes for the detection and visualization of these DNA structures in cells. Metal complexes often present advantageous photophysical properties, like their significant Stoke shifts, which can prevent self-quenching and allow for easy resolution of the excitation and emission light. Moreover, the long fluorescence lifetime of metal complexes compared to organic dyes allows their fluorescence to be readily distinguished in the presence of endogenous fluorophores, usually present in biological environments, by use of time-resolved spectroscopy (TRS) or fluorescence lifetime imaging microscopy (FLIM). Another advantage of fluorescent metal complexes is that their fluorescence can be sensitive to changes in the local environment and, therefore, their fluorescence emission can be directly modified by G4 binding. In fact, they can act as “light-up” probes that display a strong enhancement upon G4 binding and as “light-off” probes that display a decreased fluorescence upon binding. There is a third type of metal-based G4 binders that behave as permanent probes (“tagged” G4-binders), exhibiting no variation of fluorescence upon G4 binding but displaying G4 binding specificity.

Metal-based G4 binders can combine anticancer properties with fluorescent properties, which confer them with imaging/diagnostic capabilities. This combination might allow exploring the so-called “theranostic” approach based on the use of a single metal complex capable to detect the presence of G4 structures in cancer cells and tissues and, at the same time, to exert anticancer effects. Biomedical optical imaging using fluorescent probes is an emerging modality in cancer imaging, being a powerful tool for preclinical studies with different animal models and for image-guided surgery procedures [71,72]. However, it has its own limitations due to the short penetration of light and near-infrared (NIR) photons in biological tissues that limits its usefulness for in vivo whole-body applications, namely in the case of widely spread metastatic disease. Nuclear medicine imaging techniques are an attractive alternative to circumvent these difficulties, due to their high sensitivity and almost unlimited depth in tissues of the gamma photons emitted by medical radionuclides. They comprise the Single-Photon Emission Computed Tomography (SPECT) and the Positron Emission Tomography (PET), both presenting a high translational potential and being able to provide unique biological information, at molecular level, on healthy and pathological processes [71,73]. 

Nuclear medicine procedures usually involve the intravenous administration of radiolabeled drugs that are called radiopharmaceuticals, which are used for PET or SPECT diagnostic or for therapeutic applications depending on the physical properties of the labeling radionuclide. Nuclear modalities offer the unique advantage of using the same chemical entities, labelled with a diagnostic radionuclide or a therapeutic one, as theranostic radiopharmaceuticals suitable for the diagnostic, therapy, and follow-up of diverse types of cancer [74,75]. Metal complexes play a prominent role in the design of theranostic radiopharmaceuticals since there are many radioactive metals with nuclear properties adequate for imaging or therapy. Surprisingly, studies on radiometallated G4 binders are almost inexistent even if some of the metals most studied to obtain metal-based G4 binders, such as copper and platinum, present radioisotopes suitable for nuclear imaging and therapy. By contrast, several reports have been published on classical DNA intercalators labeled with medical radionuclides, including preclinical studies with cellular and animal models of cancer [76]. 

### 1.3. Scope of the Review

Here, we propose a comprehensive and critical overview of the diverse molecular design strategies that have been studied to obtain metal based G4 binders, with a focus on fluorescent probes and anticancer agents for diagnostic and/or therapy of cancer. The review covers a large variety of complexes that are presented in the next sections according to the respective ancillary ligands, spanning from Schiff bases to multidentate N-heterocyclic ligands (e.g., terpyridine, bipyridine, and phenanthroline derivatives) and to macrocyclic chelators (e.g., porphyrins and phthalocyanines). As exemplified in Figure 5, the review will focus mainly on monomeric complexes, carrying in some cases pendant G4-binding motifs. However, dimetallic complexes with homogeneous and heterogeneous metal centers and multinuclear metal assemblies will be also reviewed. Finally, it will be also discussed the pertinence to develop radiometallated G4 binders as tools for cancer theranostics, in light of the developments that were reported for classical DNA intercalators labelled with radioactive metals.

## 2. Metal-Based G4 Binders for Cancer Theranostics: Fluorescent Probes and Anticancer Agents

### 2.1. Complexes with Schiff Bases

Schiff bases, also known as imines (C = N) or azomethines (HC = N), are among the most popular ligands in coordination chemistry owing to their suitability to coordinate with many transition metal ions, providing complexes with the metal in different oxidation states that have been studied in different applications, namely as catalysts, biomimetics, and anticancer drugs. One of the most widely studied classes of Schiff bases correspond to the salen-type ligands, obtained by condensation of salicylaldehyde derivatives with aliphatic or aromatic diamines. Salen-type ligands have been extensively used to obtain complexes (metallosalens) with a wide range of metal centers including Pd, Pt, Cu, Zn, Ni, etc. Metallosalens were thoroughly studied as G4 DNA-binding agents taking advantage of the versatility of these structures, making it possible to afford different geometries and to introduce different substituents at different positions [60,77,78,79,80,81,82,83,84,85,86,87,88,89].

**Figure 6:**
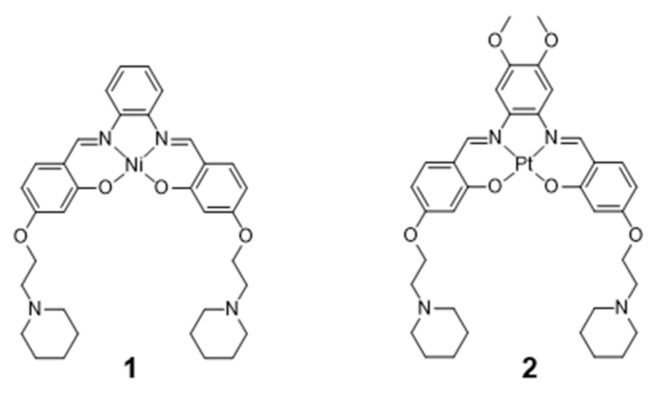
Chemical structures of square-planar M(II)-salphen (M = Ni, Pt) complexes.

The coordination geometries of metallosalens are directly dependent on the metal ion. For example, square planar geometries were reported for Ni(II), Cu(II) and Pt(II), distorted trigonal bipyramidal geometries for Zn(II) and square-base pyramidal geometries for [V=O]^2+^ [89]. Salen complexes usually bind non-covalently to DNA, either via intercalation or groove-binding, depending on the coordination geometry of the metal and the structure of the ligand [83,87,89,90]. Most relevantly, the planar π-delocalized metallosalens are well suited to stack on the face of the G4 tetrad, with the electropositive metal ion positioned at the center of the G-quartet and replacing the stabilizing K^+^ ion at the top of the stack. Moreover, the affinity of metallosalens for G4 DNA can be further enhanced by introducing pendant substituents, like DNA recognition entities or positively charged groups for additional electrostatic interactions with phosphate groups in the DNA backbone. Due to these favorable features, several metallosalens have shown good affinity towards G4 DNA (mainly human telomeric sequence (H-Telo) and *c-myc*), as for example the Ni(II) and Pt(II) complexes presented in Figure 6. Most of the G4 DNA binding constants of salen complexes fall in the 10^3^–10^7^ M^−1^ range [52]. Moreover, different metallosalens have displayed excellent selectivity for telomeric G4 over duplex DNA, as for instance the Nickel(II)–salphen complex reported by Vilar and co-workers (complex **1**, Figure 6) [60]. Complex **1** exhibits a 50-fold selectivity for G4 over duplex DNA, as shown by FRET (Fluorescence Resonance Energy Transfer) melting assays. 

In 2006, Che and co-workers reported on a series of Pt(II)-salphen derivatives that were investigated as fluorescent DNA-binding theranostic agents. For this purpose, the authors examined the complexes interaction with *c-myc* G4 DNA by means of absorption, emission and NMR titration experiments, as well as by molecular modeling [85]. The best results were obtained for complex **2** (Figure 6), which performed as an efficient G4 stabilizer. Despite presenting a low photoluminescence quantum yield (ϕ = 0.01), this complex exhibited an eightfold increase in fluorescence intensity at λ_max_ = 652 nm upon binding to *c-myc* G4. Complex **2** effectively down-regulates the expression of *c-myc* in human hepatocarcinoma cells, as shown by RT-PCR assays; it showed moderate, but significant, cytotoxicity against the same cancerous cell lines (IC_50_ = 8–14 μM) with a tenfold lower cytotoxicity towards normal lung fibroblasts cells. This selectivity makes it possible for this Pt(II) complex to effectively inhibit *c-myc* gene promoter activity at concentrations that do not have significant toxic effect in normal cells.

**Figure 7:**
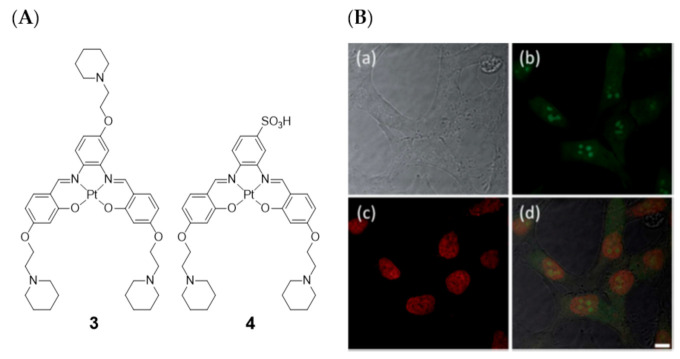
(**A**) Structures of Pt(II)–salen complexes. (**B**) Fluorescence microscopy of live HeLa cells stained with complex 3. (**a**) shows the transmitted light image, (**b**) is the fluorescence intensity image, (**c**) shows DRAQ5 nuclear staining (5 mM) and (**d**) represents the merge of the three channels. Clear fluorescence is seen in the nucleus of all cells indicating that the complex localizes in this region and becomes fluorescent in the presence of DNA. Scale bar 20 microns. Reproduced from ref. [83] with permission from The Royal Society of Chemistry.

Later on in 2014, Vilar et al. also evaluated two platinum(II)–salen complexes (complexes **3** and **4**, Figure 7) through fluorescence microscopy imaging in four different cell lines (CHO, HeLa, U2OS, and HepG2) [83]. 

These studies showed that the cell permeability of the two probes is highly dependent on the cell line used, as well as their intracellular localization. Neither of these two platinum(II) complexes were taken up by U2OS cells under the experimental conditions used. For the other three cell lines, complex **3** was readily taken up while complex **4** was only partially cell permeable after long incubation times. In HeLa cells, the less hydrophilic complex **3** exhibited a characteristic nucleus staining, localizing mainly in the nucleoli (see Figure 7B). The introduction of the negative charged sulfonate group in complex **4** aimed at a more selective G4 DNA binding compared with duplex DNA, avoiding non-specific electrostatic interactions with DNA and favoring non-covalent π–π staking DNA interactions. This could be confirmed by FRET competition assays. However, the imaging studies with fluorescence microscopy showed that complex **4** is not taken up by the cells as readily as complex **3**, probably because the sulfonate negative charge reduces the ability to cross biological membranes and, thus, hampering also the nucleus uptake.

**Figure 8:**
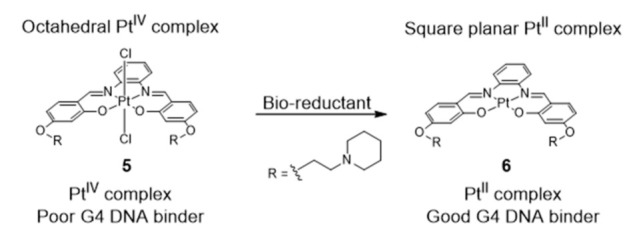
The octahedral Pt(IV) complex (**5**) does not bind to G4 DNA, but upon reduction to Pt(II) a G4 DNA binder is generated yielding the first example of a redox-triggered G4 DNA binder [84].

More recently, Vilar et al. reported a redox-activated G4 binder consisting in an in situ generated platinum (II) complex [84] (Figure 8). 

The octahedral platinum (IV)–salphen complex (**5**) does not interact with DNA in aqueous media at pH 7.4. However, upon reduction in the presence of bio-reductants (e.g., ascorbic acid and glutathione), the complex turns into an active (G4 binder) planar platinum (II) complex (Figure 8). The cytotoxicity and the optical properties of complex **5**, acting as a Pt(IV) prodrug, are, therefore, activated under reduction conditions, i.e., in tumor environments (known to be under hypoxic conditions). After activation, the reduced Pt(II) complex not only becomes cytotoxic but also becomes highly emissive upon interaction with G4 DNA, therefore, this Pt^IV^/Pt^II^ system can be easily monitored in vitro, which facilitates its use in cancer theranostics.

**Figure 9:**
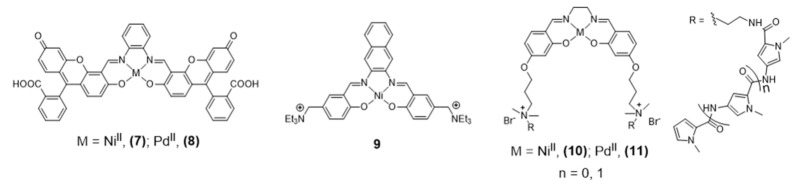
Chemical structure of Ni^II^ and Pd^II^ salphen complexes.

Ni^II^ and Pd^II^ salphen complexes **7** and **8** (Figure 9), reported by Bhattacharya [87] are also emissive thanks to the fluorescein incorporated in the ligand’s framework. 

The studies done in cancer cells HEK293T and A549 using confocal microscopy showed that these complexes are cell-permeable and accumulate in the nucleus and mitochondria. The extended aromatic surface provided by the ligand’s framework demonstrated to be adequate also for H-Telo G4 DNA binding with promising inhibition of telomerase activity. Barone et al. also reported a different Ni-salphen (**9**, Figure 9) bearing an extended aromatic framework that demonstrated good G4 binding properties. The analogue complexes obtained with Zn(II) and Cu(II) revealed less potent G4 binding stabilization and in vitro fluorescence imaging studies were not reported for these complexes [82,88].

In 2016, Bhattacharya et al. reported different novel salen-based Ni^II^ and Pd^II^ metal complexes (**10** and **11**, Figure 9) with positively charged flanking side chains comprising N-methylpyrrole carboxamides of variable lengths [87]. The conjugation of the tetrad-binding metal salen core with groove-oriented flexible oligopyrrole moieties resulted in a highly selective stabilization of human G4 DNA structures. Although complexes **10** and **11** are structurally similar with the only difference being the central metal ion, they showed different binding affinities and selectivity towards G4 DNA. The Ni(II) complex led to a greater stabilization of G4 DNA, which was justified by the smaller ionic radius of Ni^II^ compared with Pd^II^ and by the formation of additional hydrogen-bonding interactions. The cellular uptake of the complexes was not followed by fluorescence microscopy studies, but cellular staining experiments allowed for the detection of significant changes in cell morphology and nuclear integrity of HEK 293T cells treated with complexes **10** and **11**, including nuclear condensation and fragmentation in agreement with the cytotoxicity observed for these compounds. 

**Figure 10:**
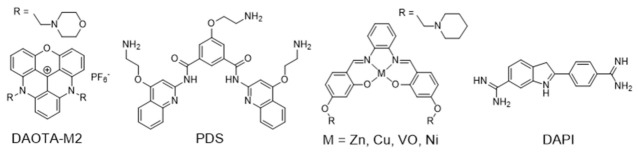
Chemical structures of DNA binders studied in a FLIM-based cellular assay [91].

A large number of metallosalens do not present fluorescent properties suitable for microscopy imaging studies with live cells. To circumvent this limitation, Vilar and co-workers developed recently a new quantitative fluorescence lifetime-based displacement assay to visualize the strength and the rates of the interaction of these small molecules with G4 in live cells [91]. This innovative assay relies on the use of the molecule DAOTA-M2 (Figure 10) that shows remarkably different fluorescence lifetime when bound to G4 structures, as compared to duplex or single stranded DNA. 

DAOTA-M2 also shows a good live cell permeability and a low cytotoxicity. These favorable features prompted its use in this new FLIM-based cellular assay, which can be applied to a wide range of drug candidates. To validate this displacement assay, the authors used pyridostatin (PDS) as a reference G4 binder, whose behavior was compared with the tested metal-salphen complexes and with DAPI as a negative control (Figure 10). DAPI localizes in the nucleus (and binds to DNA) but did not change the nuclear mean weighted average fluorescence lifetime (τw) of DAOTA-M2, consistent with DAPI not displacing the probe from G4 DNA. By contrast, PDS displaced DAOTA-M2 from G4 back to dsDNA because it acts as a highly specific G4 binder with a greater affinity for G4 than DAOTA-M2, which led to a drop in τw. The study of the DAOTA-M2 displacement upon addition of Ni-salphen, which is a G4 binder with higher affinity for G4 and dsDNA than DAOTA-M2, showed also a drop of τw to ca. 3.7 ns that is close to the fluorescence lifetime of the free dye. To confirm the usefulness of this technique a comparative study was done using a range of M-salphen complexes (M= Ni, Cu, VO, Zn), showing a different G4 binding affinity. 

### 2.2. Complexes with N-Heterocyclic Ligands

#### 2.2.1. Terpyridine, Bipyridine and Related Ligands

**Figure 11:**
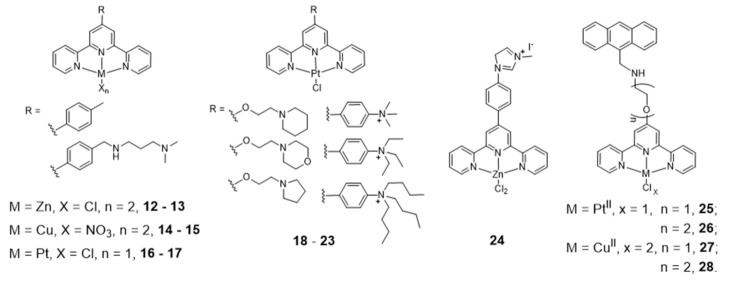
Chemical structures of terpyridine complexes with divalent metal ions.

The effect of the metal ion in the coordination geometry of several terpyridine complexes has been evaluated and correlated with G4 recognition and stabilization (Figure 11).

Teulade-Fichou et al. studied the ability of non-planar Zn(II)–terpyridine complexes (**12**–**13**) to bind G4 and concluded that these complexes were poor G4 binders compared with similar but planar Cu(II)– and Pt(II)–terpyridine complexes (**14**–**15** and **16**–**17**) [92]. Moreover, the Pt(II)–terpyridine complexes were modified with cyclic amines or peralkylated ammonium-based side chains (**18**–**23**, Figure 11), which enhanced binding affinity and selectivity for G4 structures over duplex DNA [93,94]. 

More recently, Natalia Busto et al. published a structure activity relationship (SAR) study with Zn chloride or nitrate complexes containing terpyridine ligands functionalized with different substituents [95]. The study revealed that the choice of the leaving group, Cl^-^ vs. NO_3_^-^, exerts a strong influence on the cytotoxicity of the compounds but not on the G4 thermal stabilization, being the chloride complexes more cytotoxic than the nitrate analogues. There is also an effect of the terpyridine substituents on the biological behavior of the complexes, and the presence of a methylated 4-(imidazol-1-yl)phenyl substituent provides the most stabilizing G4 ligands, probably due to their extra positive charges, namely complex **24** (Figure 11) that displayed the highest cytotoxicity in colon adenocarcinoma cells. The enhanced anticancer activity of complex **24** was correlated with its highest selectivity and affinity towards human telomeric G4 Tel22, and with its preferential localization in the cell nucleolus as shown by fluorescence microscopy assays.

**Figure 12:**
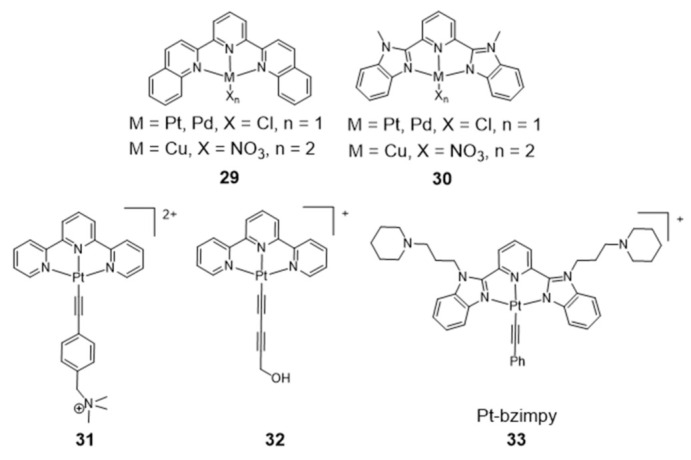
Chemical structures of M(II) (M = Pt, Pd, Cu) complexes with terpyridine, extended terpyridine and bis(imidazole)pyridine ligands.

Gama et al. also studied a small family of Cu(II)– and Pt(II)–terpyridine complexes, **25**–**28** (Figure 11), in which the terpyridine is tethered with a planar anthracene moiety via different linkers [61]. The complexes showed affinity for G4-forming sequences (H-Telo and *c-myc*) with a good selectivity over duplex DNA (ds26 and ct-DNA). Importantly, the free ligands do not have significant affinity for any of the DNA sequences studied, which shows that the presence of the metal is essential for high affinity (and selectivity). This effect is more evident in the case of the Pt(II) complexes. Moreover, the presence of a longer linker between the chelating terpyridine unit and the anthracene moiety enhances the interaction with G4 forming sequences. The [Pt(Ant-tpy)Cl]^+^ complexes (**25** and **26**) targets the G4 DNA structures more selectively than the copper analogues [Cu(Ant-tpy)Cl_2_] (**27** and **28**), despite the expected better ability of the planar [Pt(tpy)Cl]^+^ unit to intercalate between adjacent base pairs of duplex DNA if compared with the [Cu(tpy)Cl_2_] unit. The authors claimed that these results could be justified by an intricate interplay of effects such as π-stacking with the accessible G-tetrad or with loops and grooves of the G4 DNA elements, as well as electrostatic interactions involving the more polar pendant arm. The extension of the planar aromatic surface of the terpyridine or bis(imidazole)pyridine scaffolds, in complexes of type **29** and **30** (Figure 12), also enhances the binding affinity and specificity of M(II)-complexes for G4 DNA [96]. 

As shown in Figure 12, several Pt(II)–terpyridine (**31** and **32**) and terpyridine-like Pt(II) complexes (Pt-bzimpy, **33**) carrying conjugated alkynyl co-ligands were synthesized and evaluated as luminescent probes for G4 DNA imaging. The obtained results showed that this class of compounds are promising probes to study the biological functions of G4 DNA structures. For instance, Yam et al. patented in 2008 complex **31** that binds to human telomeric G4 with increase of its fluorescence at around 625 nm [97]. A similar luminescent water-soluble alkynylplatinum (II) terpyridine (complex **32**) was also reported by the same researchers for the detection of intermolecular formation of DNA G4 from unfolded DNA [98]. Che and co-workers synthesized a series of platinum(II) complexes containing a 2,6-bis-(benzimidazol-2-yl)pyridine (bzimpy) ligand [99], which exhibited tenfold or higher selectivity for binding to G4 over duplex DNA. One particular derivative, **33**, exhibited a 38-fold increase in luminescence upon G4 binding but only a fourfold increase upon saturation with calf thymus DNA. Although **33** exhibited very mild cytotoxicity in a panel of human tumor cells, no cellular fluorescence imaging studies were reported for this complex.

**Figure 13:**
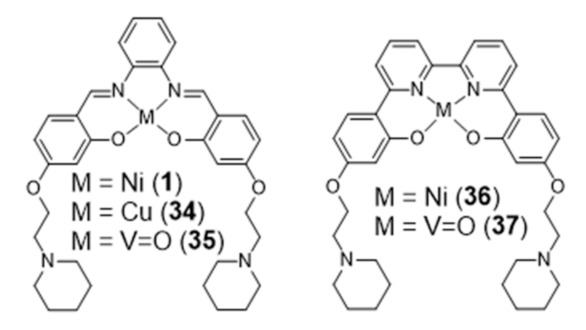
Chemical structures of Ni(II) and V^IV^=O complexes stabilized by N_2_O_2_ ligands containing a 2,2’-bipyridine group (right) and congener metal salphen complexes (left).

In 2018, Vilar et al. reported new Ni(II) and V^IV^=O complexes stabilized by N_2_O_2_ ligands that contain a 2,2’-bipyridine group, and performed a comparative study with the analogue salphen complexes already mentioned in the previous section (Figure 13) [100]. 

**Figure 14:**
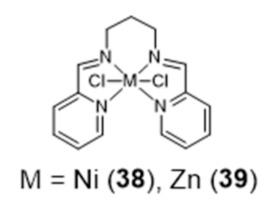
Ni^II^- and Zn^II^-complexes whose G4 DNA binding was assessed by SERS.

In this study, the selectivity of the different complexes against six different G4 DNA structures of different topology has been evaluated via FRET melting assays. The results showed that the new metal complexes **36** and **37** are better G4 DNA binders than the corresponding metal salphen complexes, displaying high affinity for G4 DNA and selectivity over duplex DNA. All of them show a preference towards antiparallel and hybrid conformations over parallel ones. Recently, Basu et al. reported an interesting contribution dealing with the possibility to detect G4 DNA through highly sensitive Raman spectroscopy, using new Zn(II) and Ni(II) complexes anchored by pyridyl-containing N_4_ ligands (**38** and **39**, Figure 14) [101]. In particular, the studies done with surface-enhanced Raman spectroscopy (SERS) showed that the complexes undergo a decrease of the Raman signal intensity upon interaction with various G4 structures, when gold nanoparticles were used as the signal enhancer to analyze such interactions. 

**Figure 15:**
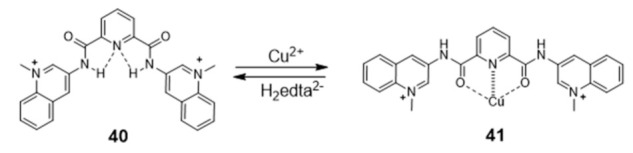
Illustration of the switch between the planar and linear conformation for a pyridyl-containing ligand, upon addition of Cu(II) or H_4_edta (ethylenediamine tetraacetic acid).

Generally, the introduction of metal ions in given molecular structures results in a stronger interaction with G4 DNA, offering a more optimal molecular geometry and cationic properties. However, Mergny and co-workers reported a clear example, based on a pyridine derivative, where the presence of the metal ion is not favorable towards G4 binding. In this study, the coordination of a selective G4-binder, the bisquinolinium ligand **40** (Figure 15) to copper(II) causes a planar-to-linear conformation transition that leads to the loss of G4 binding ability.

The most probable explanation to this loss of affinity is the breaking of the planar π–delocalized surface of the free ligand after copper complexation and disruption of intramolecular hydrogen bonds, which cause the unfolding of the G4 structure into a single strand [102].

#### 2.2.2. Phenanthroline and Related Ligands

##### Platinum Complexes

**Figure 16:**
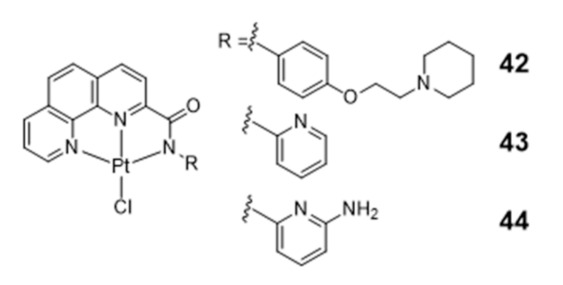
Chemical structures of Pt (II) complexes with phenanthroline ligands functionalized with piperidine (**42**) and pyridyl (**43**–**44**) pendant arms.

Several square-planar platinum(II)–phenanthroline complexes have been used successfully to target telomeric G4 DNA. For instance, phenanthroline ligands modified with a pendant cyclic amine or pyridine side arms, through a single amide link, afforded complexes **42**–**44** that exhibit high affinity for H-Telo DNA and act as moderate telomerase inhibitors (Figure 16) [59,103].

**Figure 17:**
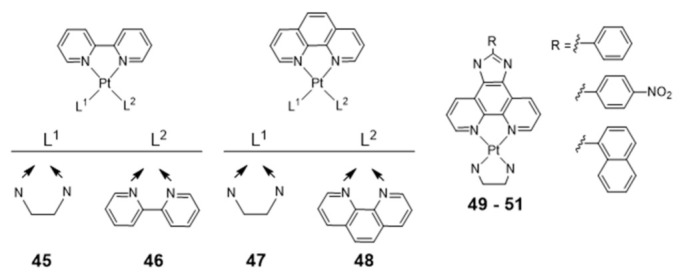
Homoleptic and heteroleptic Pt(II) complexes with aliphatic and aromatic N,N-donor ligands.

Homoleptic or heteroleptic Pt(II) complexes, stabilized by aliphatic or aromatic diamines, are another class of compounds that were investigated as potential G4 DNA binding ligands (Figure 17) [104,105,106]. Both for the homoleptic and heteroleptic systems, it was found that the phenanthroline-containing complexes (**47** and **48**) establish a stronger interaction with G4-DNA structures than the bipyridine counterparts (**45** and **46**) complexes (Figure 17) [104,105,106]. 

Complexes **49**–**51** showed a further enhancement of the selectivity and affinity towards G4 DNA with binding constants larger by two orders of magnitude, upon replacement of the phenanthroline by a phenanthroimidazole moiety that led to the presence of an extended π-delocalized surface [106,107,108,109].

**Figure 18:**
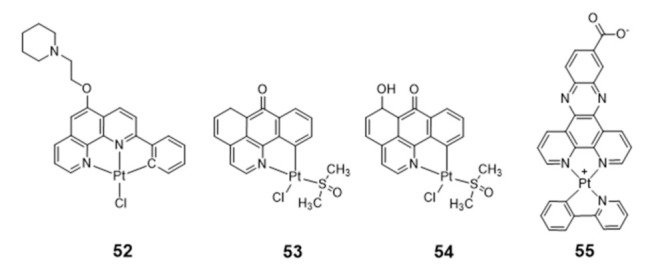
Chemical structures of organoplatinum complexes acting as G4-DNA binders.

Vilar and co-workers reported the synthesis of a new phenanthroline-based ligand and its corresponding cyclometallated platinum(II) complex (**52**) (Figure 18) [110]. This organoplatinum complex interacts selectively with G4 DNA with consequent “switch-on” of its fluorescence emission properties.

Complex **52** alone is not taken up by osteosarcoma U2OS cells, which prompted its encapsulation inside a hexaruthenium cage to promote the transport of the complex into these tumoral cells and the follow-up of its localization and interaction with cellular targets by optical imaging studies. Emission studies of complex **52** in the presence of different G4 DNA sequences showed that the emission enhancement was much more pronounced in the presence of *c-myc* G4 and a ribosomal G4 DNA (6183 NT) than in the presence of duplex DNA, underlying the suitability of this complex for the selective detection of G4 structures over duplex DNA. Moreover, co-staining experiments in U2OS cells revealed that the complex localization did not overlap with that of the duplex DNA probe DAPI. Unlike DAPI, complex **52** accumulates mostly in the nucleoli, which suggested that it may target alternative DNA topologies in the cells such as G4 DNA.

Liang reported two organoplatinum complexes (**53** and **54**, Figure 18) containing both a labile chloride ligand and a N,C-donor ligand showing a π-conjugated planar aromatic system, which allowed platination of the guanine nucleobases of G4 DNA and non-covalent π-stacking with the G-quartets [111]. Complexes **53** and **54** were tested in a tumor xenograft mouse model, displaying a low systemic toxicity with in vivo antitumoral effect greater than cisplatin, emerging, therefore, as relevant compounds for the development of anticancer drugs. Both complexes act as telomerase inhibitor targeting G4 DNA. However, **54** showed a stronger telomerase inhibition ability and a better selectivity for G4-DNA. The authors attributed these differences to the presence of a 6-hydroxyl group in the ligand of complex **54**.

Organoplatinum(II)–dipyridophenazine complexes usually exhibit excellent photophysical properties. Having this in mind, Ma et al. synthesized a series of Pt(II) complexes with dipyridophenazine (dppz) and C-deprotonated 2-phenylpyridine(N^CH) ligands, and evaluated them as telomerase inhibitors and G4 binders [112]. The series of tested compounds included complex **55** containing the aromatic dppz scaffold functionalized with a pendant COOH group (Figure 18). Complex **55** showed a 293-fold increase in photoluminescence upon G4 binding, a 10-fold binding selectivity for G4 over duplex DNA and a submicromolar inhibition potency against telomerase (IC_50_ = 760 nM). Moreover, the compound showed a notorious cytotoxicity against multidrug- and cisplatin-resistant cell lines, being 10-fold less cytotoxic to normal lung fibroblast cells. Altogether, these results were justified by the presence of the extended aromatic dppz scaffold, enabling complex **55** to effectively interact with the terminal face of human telomeric G4 through end-stacking interactions, and by the presence of the pendant COOH group favoring H-bonding interactions with the guanine residues of the external G4-tetrads. 

##### Ruthenium Complexes

**Figure 19:**
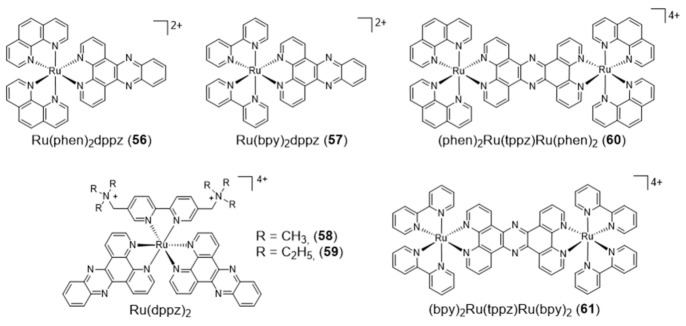
Chemical structures of ruthenium complexes containing dppz and related ligands.

As shown in Figure 19, the heterocyclic aromatic dppz ligand has been also used to obtain several octahedral ruthenium complexes, acting as DNA binders and presenting interesting photophysical and electrochemical properties [113].

In these complexes, the metal is not part of a planar unit with DNA intercalating properties and will interact with the grooves and loops of G4 DNA structures rather than with the G-tetrad. For instance, Shi and co-workers reported the complex [Ru(phen)_2_dppz] (**56**), which does not emit luminescence in aqueous solution upon photoexcitation of the metal-to-ligand charge transfer (MLCT) transition. However, complex **56** exhibited fivefold higher luminescence when bound to G4 DNA relative to i-motif DNA, which corresponds to four-stranded secondary structures formed in sequences rich in cytosine [114]. The same authors also reported that the congener complex with bipyridine ([Ru(bpy)_2_dppz] (**57**)) could differentially bind various G4 topologies, being able to bind the G4 of H-Telo DNA, with high affinity and a significant luminescence response [115]. Furthermore, it was found that the complex could induce the formation of the G4 motif even in the absence of stabilizing cations. Mao and Ji synthesized two new tetracationic ruthenium complexes, each containing two dppz ligands and an alkylammonium bipyridine ligand (**58** and **59**). These complexes bound to G4 DNA with binding constant values of 9 × 10^7^ and 4.5 × 10^7^ M^−1^, respectively. Upon binding to G4 DNA, luminescence increased approximately five- and eightfold for **58** and **59**, respectively [116]. 

Thomas and co-workers reported two dinuclear polypyridylruthenium complexes, [(Ru(phen)_2_)_2_(tppz)]^4+^ (**60**) and [(Ru(bpy)_2_)_2_(tppz)]^4+^ (**61**) where tppz=tetrapyridophenazine (Figure 19). These dinuclear complexes display strong binding affinities to both duplex DNA and antiparallel G4s formed from H-Telo DNA [117]. In fact, in vitro studies demonstrated that **60** bounds to G4s with affinity (K_d_ = 0.23 μM) very similar to that for duplex DNA (Kd = 0.32 μM) [117]. Interestingly, the interaction of these complexes with H-Telo was accompanied by a 150-fold increase in the luminescence signal, with a blue shift of approximately 30 nm of the emission maximum. The enhancement observed upon interaction with G4 H-Telo is considerably more intense relative to duplex binding. It was also verified that complex **60** could selectively stain the nuclei of live MCF-7 cells when applied at 500 μM [118].

**Figure 20:**
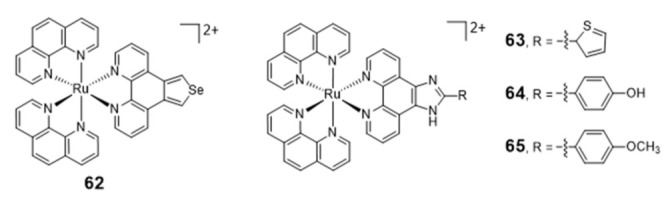
Ruthenium(II) complexes that recognize telomeric DNA and inhibit telomerase activity.

Liu et al. reported a series of [Ru(phen)_2_(N^N)] complexes (**62**–**65**, Figure 20) with modified phenanthroline ligands, e.g., containing imidazole or selenazole rings, and studied their usefulness as luminescent light switches for the detection of telomeric DNA [119,120,121,122].

These complexes were able to bind to the H-Telo G4 with strong affinities and a significant enhancement of the luminescence, showing also promising telomerase-inhibition activities. Interestingly, some complexes displayed a broad spectrum of cytotoxicity against human cancer cells, while remaining inactive (IC_50_ > 100 μM) towards a normal cell line. The high luminescence and photostability of complexes **64** and **65** allowed cellular imaging studies that provided spatio-temporal information on its intracellular localization in live Hep G2 cells and proved their ability to accumulate in the nuclei [119,120]. 

Chen et al. reported a new family of water soluble ruthenium(II) complexes with three chiral ligands: R/S-(±)-4-(2,3-dihydroxypropyl)-formamide oxoaporphine (R/S-(±)-FOA), R-(+)-4-(2,3-dihydroxypropyl)-formamide oxoaporphine (R-(+)-FOA), and S-(−)-4-(2,3-dihydroxypropyl)formamide oxoaporphine (S-(−)-FOA) [123]. These Ru(II) complexes exhibited strong affinity to G4-DNA and caused inhibitory effects on the telomerase activity, which are significantly correlated with the chiral nature of the ligands. The in vivo experiments in tumor bearing mice showed that the treatment of the animals with one of the chiral complexes led to a significant reduction in tumor size, thus, emerging as a promising candidate for the development of more effective anticancer agents.

**Figure 21:**
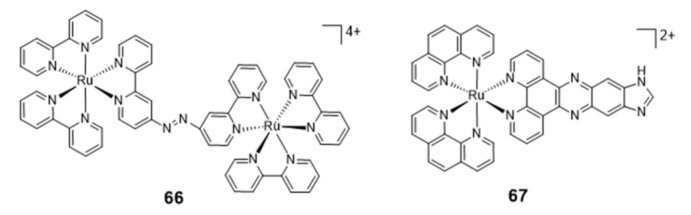
Selected examples of ruthenium complexes patented as DNA G4 probes.

The success obtained with ruthenium complexes for the stabilization of G4s made it possible to patent some of them, namely a series of ruthenium chelates bearing an azo bridging group and acting as DNA groove binders and a series of mononuclear ruthenium derivatives carrying extended dppz ligands [124,125]. Among these compounds, the dinuclear Ru complex **66** (Figure 21) binds to G4 with a red shift of its UV maximum absorption band from 544 to 579 nm, but it seems that its recognition of G4s is not selective due to the strong binding to A-rich sequences. 

### 2.3. Complexes with Macrocyclic Ligands

#### 2.3.1. Porphyrin and Phthalocyanine Derivatives

**Figure 22:**
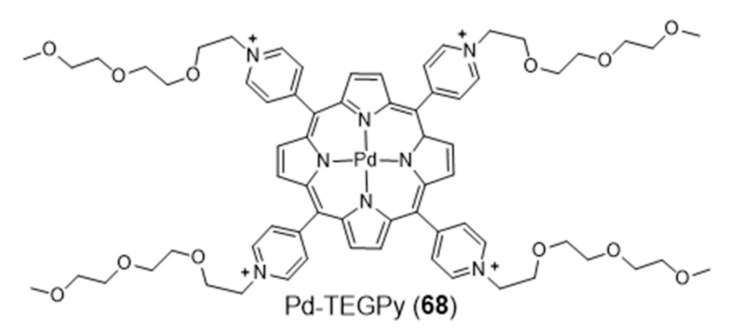
Chemical structure of Pd-TEGPy.

Metalloporphyrins have been extensively studied as G4 binders, since these complexes have the right size, symmetry, and geometry to stack onto the G-tetrad through π-π interactions. Searching for a G4-selective fluorescent dye, Monchaud et al. studied the complex Pd-TEGPy (**68**) (Figure 22) [126]. Complex **68** behaves as an efficient turn-on G4-selective fluorescent stain thanks to a DNA-mediated sensitization mechanism that ensures a high level of specificity, being a promising molecular tool for in vitro detection of G4 structures. In particular, it shows an amphiphilic character and discotic shape that make it prone to self-assembling in solution, with consequent enhancement of selectivity for G4s.

Different cationic porphyrins complexes were also studied by Pattanayak et al. as potential KRAS G4 stabilizing ligands, using molecular modeling and docking studies [127]. From this study, two novel Co and Pd porphyrin complexes emerged as potent KRAS-promoter/G4 binders. The efficacy of these ligands against human pancreatic ductal carcinoma cell lines (PANC-1 and MiaPaCa2) was evaluated, as the KRAS mutation is prevalent in pancreatic cancer. Both complexes exhibited significant cytotoxicity and blocked metastasis by inhibiting the epithelial to mesenchymal transition. Moreover, the expression of KRAS gene in porphyrin-treated PANC-1, MiaPaCa2 and tumor-derived EAC cells was drastically reduced, at both protein and RNA levels. In vivo studies in mice models confirmed that the compounds are effective against EAC solid tumors along with significantly low systemic toxicity.

**Figure 23:**
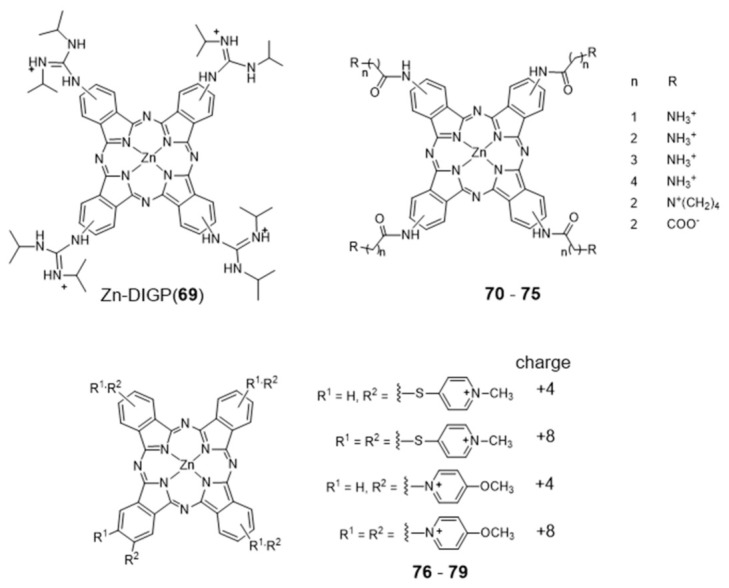
Chemical structures of Zn phthalocyanines studied as G4-binders.

Metal phthalocyanines are another broad class of metal-macrocycle complexes that have been studied as G4 binders. The zinc(II) complex Zn-DIGP (**69**) stabilized by a isopropylguanidinium-substituted phthalocyanine is a relevant example of this family of complexes (Figure 23) [128].

Complex **69** has been described by Luedtke’s group as a turn-on photoluminescent probe for *c-myc* G4 imaging, which acts also as a down-regulator of *c-myc* expression [128,129]. In a different study, it was shown that this complex stabilizes both parallel and antiparallel G4 DNA structures [129]. In addition, it was also demonstrated both in vitro and in live cells that **69** can also stabilize the G4 structure formed in the promoter region of the KRAS oncogene, and potentially regulate its expression. Complex **69** presents several advantages when compared with other analogous ammonium-containing compounds, such as water solubility and increased cellular uptake and RNA/DNA affinity [130,131]. In the presence of saturating amounts of nucleic acids, this complex undergoes a large fluorescence enhancement (200-fold, λ_ex_ = 620 nm, λ_max_ = 705 nm) but with modest fluorescence quantum yields (ϕ_F_ = 0.06). However, these low quantum yields are counterbalanced by very large molar extinction coefficients (ε = 30,000–130,000 cm^−1^ M^−1^) that result in a strong brightness (ϕF x ε), the relevant figure of merit for imaging. Remarkably, when interacting with *c-myc* G4 DNA the dissociation constant found for **69** is only 2 nM, with a 2:1 stoichiometry and a preference of one order of magnitude over unfolded G-rich ss DNA, and 1000-fold, 100-fold, and 5000-fold selectivity over C-rich unfolded ss DNA, tRNA, and calf thymus DNA, respectively. Altogether, these results highlighted that cationic metallophthalocyanines are relevant compounds for the development of highly selective luminescent therapeutics that can specifically target oncogenes because of their enhanced selectivity for the G4 compared to duplex DNA, the predominant nucleic acid conformation in the cellular nucleus.

Luedtke et al. reported on a series of Zn(II)–phthalocyanine complexes (**70**–**75**, Figure 23) functionalized with several acylamino substituents, with a systematic variation of its alkyl chain length and nature of the terminal function [132]. Complexes **70**–**75** demonstrated excellent affinity and specificity towards G4 DNA with apparent dissociation constants (K_D_) ranging from 20 to 200 nM for several G4s structures (*c-myc*, c-kit promoters and H-Telo), and showed a binding affinity for G4s 100–1000 times higher than that for duplex and single-stranded nucleic acids. In the presence of H-Telo G4, a large fluorescence enhancement was observed with **71**–**74** (100–400 fold) but not with **70** and **75**. A possible explanation for this trend is the tendency of **70** to form “J-type” aggregates (side-by-side) that are unable to bind G4 DNA, whereas **71**–**74** form “H-type” (face-to-face) dimers/multimers that are available for DNA binding. This difference is certainly due to the presence of longer methylenic linkers in the case of **71**–**74**, between the phthalocyanine core and the pendant ammonium groups, which increase the pKa values of the later. 

**Figure 24:**
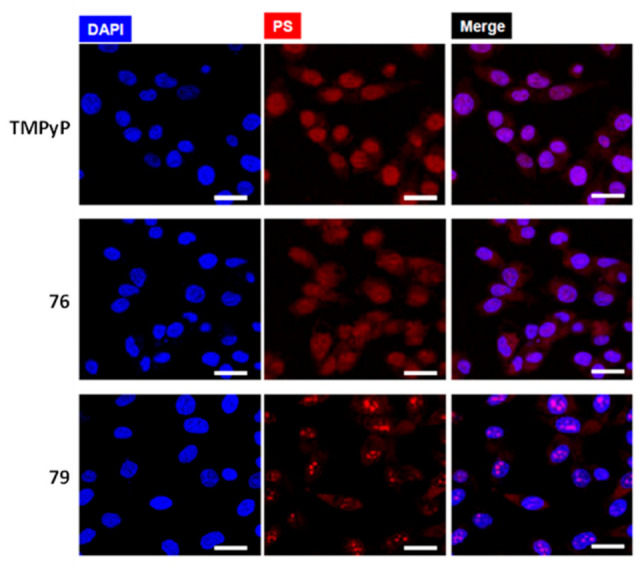
Representative fluorescence images of UM-UC-3 bladder cancer cells incubated with the ligands (red) TMPyP (30 μM), **76** (20 μM), or **79** (45 μM) for 48 h of incubation. DAPI is staining the nucleus. Scale bars 20 μm. Reproduced from ref. [133].

More recently, Neves et al. studied a series of multicharged phthalocyanines, bearing four or eight positive charges, as G4 stabilizing ligands (**76**–**79**, Figure 23) [133]. Based on this family of complexes, the authors established structure-activity relationships (SAR) to clarify the importance of both the number and the position of positive charges in the affinity and selectivity towards G4 structures. Fluorimetric titration experiments suggested a ligand: G4 binding stoichiometry of 2:1 for all the tested compounds, most likely by end-stacking interactions with the top and bottom tetrads of the G4. Complex **78**, having the four positive charges less exposed and closer to the phthalocyanine core, showed a low affinity for DNA structures. By contrast, **79** with the highest number of charges (+8) but with the same pattern of charge distribution, presented a high affinity for G4 with selectivity over duplex structures. Complex **77**, with the eight charges peripherally localized, showed identical affinity for all DNA topologies tested, being, therefore, the less specific for G4 binding. Compounds **76** and **79** presented the best affinity and selectivity to G4 structures, and their uptake in UM-UC-3 bladder cancer cells was studied by fluorescence microscopy and co-staining experiments. For both complexes, an intense emission localized in the nucleus of cancer cells was observed (Figure 24). 

#### 2.3.2. Macrocyclic-Based Metal Complexes with Pendant G4-Binding Motifs

**Figure 25:**
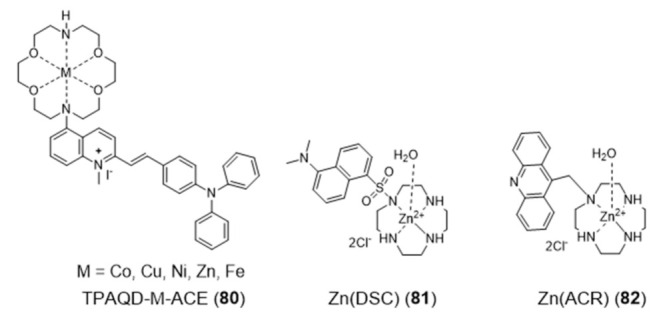
Molecular structures of macrocyclic complexes with divalent metals functionalized with pendant aromatic groups for G4-DNA binding.

Searching for new fluorescent probes for cancer cell imaging and selective targeting of G4s, Li and collaborators reported an aza-crown ether modified with a triphenylamine quinoline derivative (TPAQD-ACE (**80**)), which showed different fluorescent signal intensities upon coordination of different divalent metal ions (Figure 25), as well as different DNA binding properties [134].

The Ni(II) and Fe(II) complexes with TPAQD-ACE gave fluorescent signals at around 640 nm, in neutral buffer solutions, and exhibited excellent selectivity characteristics in the presence of different types of DNA structures. These complexes could not only efficiently distinguish G4 DNAs from single-stranded and double stranded DNAs, but also could specifically recognize the *c-myc* and long human telomeric (Hum45) G4 structures. Their G4 DNA binding characteristics were also studied in detail by molecular docking simulations. The molecular docking results corroborated that the introduction of the metal ions greatly increases the fluorescent signal intensity of TPAQD-ACE and enhances its binding ability for G4 DNAs. 

Within the design of macrocyclic complexes as G4 recognition agents, Morrow and Fountain reported two Zn(II)–cyclen complexes, **81** and **82** (Figure 25), functionalized with a nonplanar dansyl group and an acridine group, respectively, to provide G4 binding ability [135,136,137]. Complex **81** shows 110-fold selectivity in binding to H-Telo G4 over duplex DNA, evidenced by an increase in the fluorescence and a simultaneous shift in emission upon G4 interaction. The interaction of **81** with DNA was characterized by calorimetric and spectroscopic techniques, which showed a complex/G4 stoichiometry of 2:1 and indicated that there is one molecule of complex binding to two spaced thymines, within two separate loops in the H-Telo G4. Interestingly, this is the first reported metal complex-based H-Telo G4-ligand utilizing the thymine residues as the primary mode of recognition. By contrast, **82** indiscriminately binds to both H-Telo and duplex DNA.

**Figure 26:**
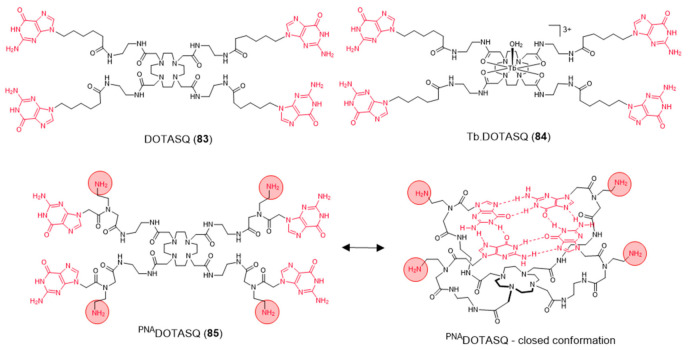
Chemical structures of DOTASQ, Tb-^PNA^DOTASQ complex, and ^PNA^DOTASQ, a smart G4 ligand, in open and close conformations (bottom).

Aiming to achieve a better control on the selectivity of G4 vs. duplex-DNA binding, Monchaud et al. designed a DOTA derivative (DOTASQ) functionalized with guanine pendant arms whose structure can rearrange when interacting with a G4 but not with duplex DNA (Figure 26) [138].

The design concept of this molecule relied on a nature-mimicking process with formation of a DOTA templated synthetic G-quartet, based on a *“like likes like”* association between two G-quartets, one native (G4) and the other artificial (ligand). However, DOTASQ (**83**) itself was unable to bind to G4-DNA, because its open conformation is dominant in solution. This difficulty could be circumvented by forming a Tb complex, Tb-DOTASQ (**84**), since metal coordination favored the DOTASQ closed conformation. Interestingly, FRET melting assays performed for the congener ligand ^PNA^DOTASQ (**85**), displaying protonable aliphatic amines adjacent to the guanine groups, showed an effective binding to G4 that is not dependent of the presence of the metal ion (Figure 26). However, the incorporation of the Tb^3+^ metal ion in the ^PNA^DOTASQ cavity compromised its G4-selectivity. This behavior was justified by random (nonspecific) electrostatic interactions between the highly cationic complex and the negatively charged DNA, whatever its nature (duplex or G4).

### 2.4. Other Metal Complexes Covalently Conjugated to G4 Binders 

**Figure 27:**
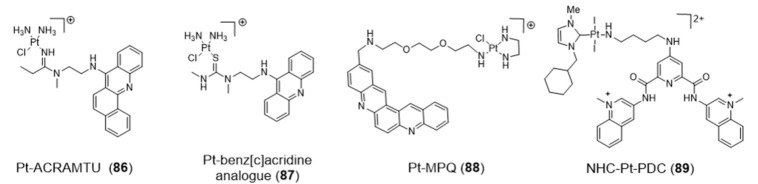
Representative examples of Pt(II) complexes functionalized with G4 binders, which can also directly platinate G4 DNA.

The conjugation of metal complexes with bioactive organic moieties has been shown to be a viable strategy to improve delivery and specific localization of novel anticancer and diagnostic tools. Specific localization or targeting may be achieved by covalent attachment of different bioactive molecules to the metal complexes, as for example G4 ligands. As shown in Figure 27, this strategy has been applied to a variety of Pt(II) complexes using different G4-ligands. 

Similar to cisplatin, most of these compounds also contain labile groups (e.g., Cl or I) and can coordinate directly to G4 DNA nucleobases, in most cases to the N7 atom of guanines through the platinum center. This type of coordination to DNA is traditionally denoted as platination and can occur either at a single-site or lead to cross-linking of two nucleobases.

**Figure 28:**
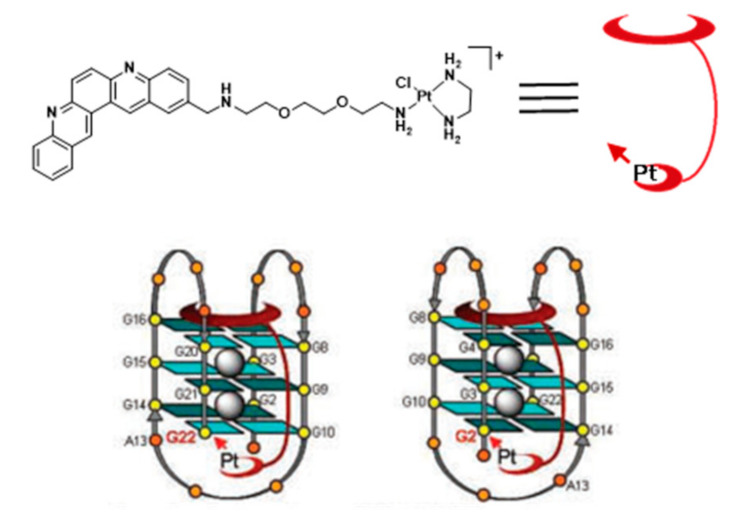
Proposed model of the interaction of Pt-MPQ (**88**) with 22AG G4. Reproduced with permission from ref. [142], published by Oxford University Press.

For example, Bierbach et al. reported several Pt(II)-acridine compounds presenting high potency against chemoresistant non-small lung cancer cells through the cross linking of DNA (see e.g., compounds Pt-ACRAMTU (**86**) and Pt-benz[c]acridine analogue (**87**), Figure 27) [139,140]. The motif ACRAMTU itself binds and stabilizes G4 DNA (∆Tm _~_13 °C in G4-22 [140]), but it also acts as a groove binder and an adenine-affinic intercalator of dsDNA [141]. Afterward, the same authors introduced a new family of platinum(II)–acridine conjugates with extended aromatic systems, studied their interaction with dsDNA and G4-DNA and evaluated their anticancer activity in a range of lung cancer cell lines. In this study, Pt-benz[c]acridine (**87**) emerged as a less genotoxic, more tolerable and potentially more target-selective hybrid agent, when compared with Pt-ACRAMTU (**86**). Complex **87** undergoes lysosomal accumulation in the tumor cells, as shown by confocal microscopy. Combined circular dichroism (CD) and HR-MS data confirmed that **87** forms coordinative adducts with the G4 structures in which the planar chromophore is π-stacked with the guanine tetrads. Bombard and collaborators described a related platinum complex, Pt-MPQ (**88**), which also interacts with G4-DNA through a double noncovalent/covalent binding mode due to the concomitant presence of the quinacridine unit and the Pt moiety, as illustrated in Figure 28.

The interaction of Pt-MPQ (**88**) with G4 DNA occurs preferentially with guanines of external G-quartets and involves a synergism between the π-stacking-directed association and the covalent trapping of the mono-functional Pt moiety, which opened new perspectives for the development of novel G4 binders [142,143]. In the same vein, the same group reported a mixed ligand Pt(II) complex (NHC-Pt-PDC (**89**), Figure 27) comprising a N-heterocyclic carbene ligand and a pendant pyridodicarboxamide (PDC) group as a G4-binding moiety. 

The NHC-Pt-PDC (**89**) complex has been successfully designed as a cytotoxic drug for telomere-based anticancer therapy, binding preferentially and irreversibly the G4 form of the human telomeric sequence. The observed covalent binding profile is different from that exhibited by NHC-Pt congeners without the PDC group, thereby indicating that the platination reaction is oriented by stacking of the PDC moiety onto the G4-structure [144].

**Figure 29:**
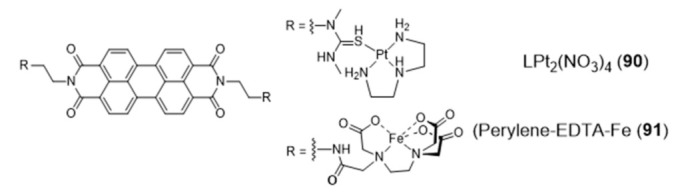
Chemical structures of perylene-containing M(II) complexes evaluated as G4 ligands.

The design of a new class of Pt(II) complexes containing suitable extended polyaromatic carrier systems, complementary to the dimensions of a G-tetrad rather than a Watson-Crick base pair, was done based on a perylenediimide bridging ligand and afforded the dimeric complex LPt_2_(NO_3_)_4_ (**90**) (Figure 29) stabilized by diethylenetriamine (dien) [145].

The interaction of complex **90** with DNA was characterized by a combination of spectroscopic and biophysical methods, which demonstrated its distinct preference for G4s exhibiting an antiparallel strand orientation (binding affinity Kb > 10^−8^ M^−1^). In the same way, a perylene derivative was also attached, on either sides, to iron(II)–EDTA complexes through the use of flexible linkers [146,147]. The resulting conjugate, perylene–EDTA–Fe(II) (**91**) (Figure 29), can interact with opposing grooves when the perylene core end-stacks on a G4-tetrad, due to its symmetric and dimeric structure. Complex **91** cleaves G4 DNA in the presence of the reducing agent dithiothreitol (DTT), acting as an artificially DNA nuclease and being the first example of a DNA-cleaving reagent of its kind with a strong affinity and selectivity for G4 DNA. 

**Figure 30:**
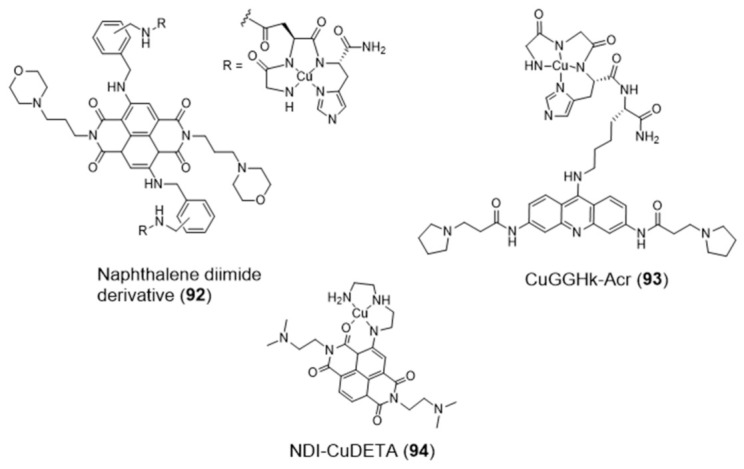
Cu(II) complexes with tetradentate chelators carrying well-known G4 binders.

The amino-terminal copper/nickel binding motif (ATCUN) can perform as a N4-tetradentate chelator forming copper-ACTUN complexes that are redox active in 3+/2+ states and can promote DNA cleavage under physiologically relevant conditions. Aiming at positioning the copper-ACTUN unit in close proximity to G4 telomeric DNA, to promote its selective cleavage, Cowan et al. reported two Cu(II)–ACTUN complexes (**92** and **93**, Figure 30) which were attached to G4-binders of the naphthalene diimide [148] or acridine types [149], respectively. It was found that both complexes act as selective nucleases of G4 telomeric DNA. Moreover, compound **92** overcame the issue of low cellular membrane permeability of traditional nucleases and the problem of slow telomere reduction commonly found for telomerase inhibitors. 

As shown in Figure 30, Freccero et al. used a different strategy to obtain a Cu(II) complex (NDI–CuDETA (**94**)) containing a water soluble naphthalene diimide (NDI) derivative. This strategy relied on the embedment of the copper complex into the structure of this G4-binder, using dien as a chelating unit that was appended at one of the NDI’s aromatic rings [150]. Complex **94** acts as a G4-cleaving agent that targets selected G4 structures, with unexpected site selectivity. The compound is stable when bound to G4 DNA structures and produce hydroxyl radicals, both in the presence and absence of H_2_O_2_, in close proximity to the Cu coordination sphere. In contrast to non site-selective DNA cleavers, the compound is highly selective for G4s (in particular the LTR-III-G4 present in HIV-1) and the hydroxyl radicals react on the target without diffusing in solution.

**Figure 31:**
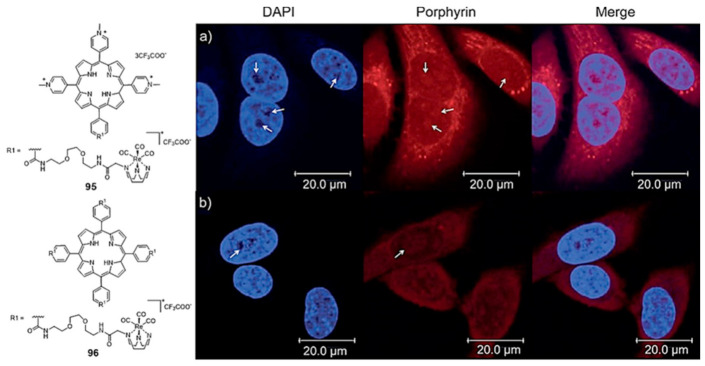
Fluorescence confocal microscopy images of HeLa cells incubated for 2 h with tricarbonyl Re(I) complexes (**95** (**a**) and **96** (**b**)) attached to a porphyrin scaffold and stained with DAPI. White arrows indicate the nucleoli. Reproduced with permission from ref. [151], published by John Wiley and Sons.

Mion et al. reported a series of Re(I)–tricarbonyl complexes containing the facially coordinated [1,4,7]-triazacyclononane (TACN) ligand, which was covalently linked to a porphyrin scaffold that can act as a photosensitizer (PS) and also as a π-stacking core to achieve G4 selectivity [151]. As exemplified in Figure 31, a variety of compounds containing a different number (n = 1 or 4) of organometallic units per porphyrin molecule was synthesized. Some of them displayed selectivity for G4 DNA (*H-Telo* and *c-myc*) over duplex DNA and a moderate fluorescence switch-on response upon G4 binding (e.g., **95** and **96**, Figure 31). The inherent fluorescence properties of these new rhenium conjugates prompted their study as PSs in photodynamic therapy and as fluorescent probes to assess the respective intracellular distribution by fluorescence confocal microscopy (Figure 31), being observed very good phototoxic indexes (PIs) for the fourfold-symmetric conjugate (complex **96)**. 

### 2.5. Multinuclear Metal Assemblies and Dimetallic Complexes

**Figure 32:**
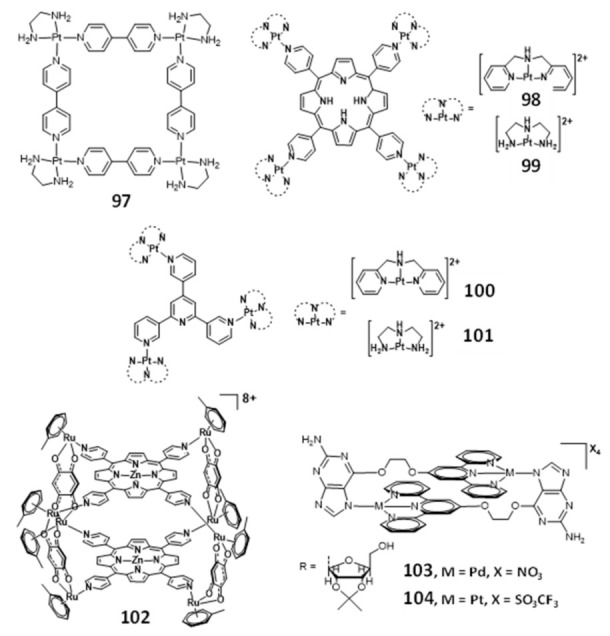
Selected examples of multimetallic complexes reported as selective G4-ligands.

Multinuclear platinum complexes are a quite relevant class of G4-ligands and several examples of platinum(II) metallo-squares are reported in the literature as effective G4 binders [152,153,154,155,156]. In fact, since the discovery of the [Pt(en)(4,4’-dipyridyl)]_4_ (**97**, Figure 32) in 2008, as the first example of a Pt(II)-square complex [152], a series of multinuclear metal assemblies have been rationally designed as effective G4-ligands.

The square arrangement of the four {Pt(en)]^2+^ at the corners and the bridging ligands of complex **97** provided a flat surface for the effective end-staking to the terminal G-quartet. In addition, the highly positive charged structure enhanced the strong electrostatic interactions with the DNA backbone, which contributed for its selectivity and efficacy as a telomerase inhibitor (IC_50_ = 0.2 μM). However, the interaction of a particular molecular assembly with G4 DNA might involve a combination of non-covalent interactions, generally through π–π end-stacking, groove binding or loop binding, depending on the geometry of the molecular assembly. 

Mao and co-workers reported several examples of multinuclear Pt(II) complexes (Figure 32), namely those exhibiting clover-like (e.g., compounds **98** and **99**) and star-like (e.g., compounds **100** and **101**) arrangements, which interact with H-Telo G4 with high selectivity over duplex DNA [157,158]. The clover-like compounds showed excellent anticancer activity as a result of a dual effect, the inhibition of telomerase activity and repression of oncogene expression [159]. Complex **101** revealed to be an attractive photosensitizer in photodynamic therapy since it accumulates in the nucleus of HeLa cells and exhibits very low cytotoxicity in the absence of light irradiation, displaying, however, a remarkable increase in cytotoxicity upon visible light irradiation [160].

Metal assemblies containing two different metals have also been studied as G4-binders, as shown in Figure 32. For example, the octacationic metallocube **102** composed of two tetraporphyrins (with or without Zn(II)) that are bridged by 2,5-dihydroxy-1,4-benzoquinato ligands coordinated to Ru^III^ centers, binds strongly to both H-Telo and *c-myc* G4 DNA but with low selectivity over duplex DNA [161]. Probably, the high cationic charge might promote non-specific electrostatic interactions with any DNA structure. Another study reported by Vilar and co-workers contributed with two novel [2 + 2] metallo-assemblies (**103** and **104**) based on a guanosine-substituted terpyridine ligand coordinated to palladium(II) and platinum(II), respectively [162]. Both assemblies interact selectively with G4 DNA (*H-Telo* and *c-myc* G4 DNA) over duplex DNA and are able to induce dimerization of parallel G4 structures, which could probably happen due to the two spaced planar “faces” of the metallo-assemblies.

**Figure 33:**
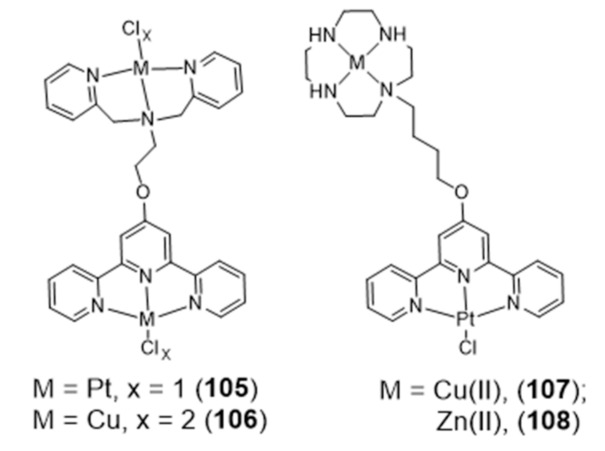
Chemical structures of dimetallic complexes with G4 binding selectivity.

In addition to the multinuclear Pt and Ru assemblies mentioned above, several bimetallic terpyridine-containing complexes with either homogeneous or heterogeneous metal centres (**105**–**108**) were reported by Vilar and co-workers and investigated as G4 binders (Figure 33). 

The strategy used to obtain these dimetallic complexes relied on the functionalization of square planar metal-terpyridine complexes, acting as G4-binders as mentioned in previous Section 2.2, with a second chelator suitable to coordinate Pt(II) or Cu(II) [163]. This strategy led to complexes with enhanced G4 binding and selectivity over duplex DNA, especially complexes **107** and **108** that presented a 1000-fold selectivity over ct-DNA. 

**Figure 34:**
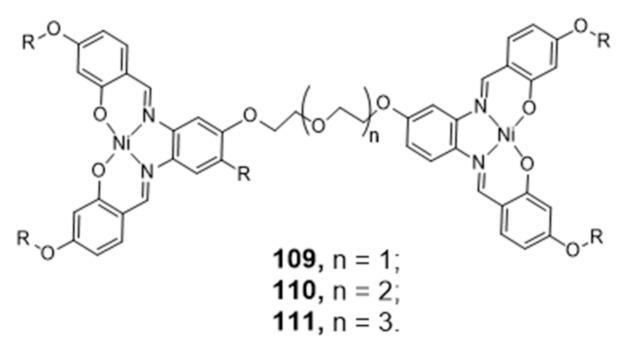
Chemical structure of di-nickel complexes designed to interact with dimeric G4s.

Following the previous successful performance of Ni-Salphen complexes as G4 selective binders [60], Vilar’s group introduced new di-metal-salphen complexes consisting on square planar Ni-salphen units bridged by polyether-tethered linkers of distinct size (Figure 34) [163]. The dinickel–salphen complex with the longest polyether linker (**111**) showed higher binding affinity and selectivity towards dimeric G4s (over monomeric G4s) than the counterparts with the shortest polyether linkers (**109** and **110**) [163]. 

## 3. DNA-Targeted Radiocomplexes

The design of G4 binders using medical radiometals can be considered an attractive approach to explore this type of DNA-targeted molecules in cancer theranostics. For this purpose, a plethora of radiometals suitable for SPECT is available (e.g., ^99m^Tc “*m*” stands for metastable, ^67^Ga, ^111^In) or PET (e.g., ^68^Ga, ^89^Zr) imaging and for systemic targeted radionuclide therapy (e.g., ^90^Y, ^177^Lu, ^225^Ac). These nuclear medicine modalities have already made an enormous contribution to the diagnosis and treatment of cancer in the clinical onset, based on the use of appropriate radioactive compounds designated as radiopharmaceuticals. For imaging, the low detection limits of nuclear modalities allow the in vivo visualization of biomarkers at low local concentration, while for therapy they are potentially useful to eradicate disseminated tumor cells and small metastasis. 

**Figure 35:**
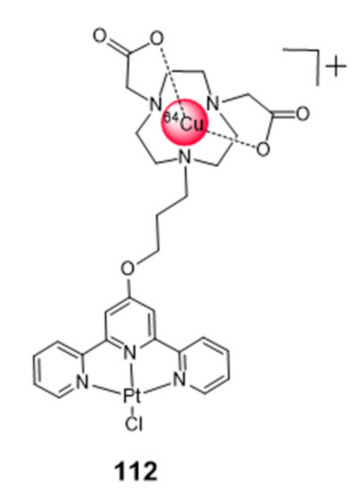
^64^Cu-labeled dimetallic NOTA-terpyridine platinum complex tested as a G4-binder.

To our view, the design of bifunctional (radio)metal chelates conjugated with G4 binding molecules could bring excellent opportunities to design new tools for cancer theranostics, by coupling nuclear imaging to specific therapeutic modalities (radio or chemotherapy). Most relevantly, as mentioned in the introductory section, copper and platinum, which are among the most studied metals to obtain coordination complexes with G4 binding properties, present different radioisotopes suitable for medical use, such as ^64^Cu, ^67^Cu or ^195m^Pt [75]_._ Besides the intrinsic interest of these radioisotopes for imaging and therapeutic use, their radiocomplexes can be seen as tracers of the corresponding compounds with the natural metals (^nat^Cu and ^nat^Pt, “*nat*” stands for natural), useful to speed-up their preclinical evaluation, namely in terms of biodistribution and pharmacokinetics studies. Despite these advantages, the examples of G4-binders incorporating a radiometal are scarce. To our knowledge, they are restricted to the work of Sanche et al., who reported a ^64^Cu-NOTA-terpyridine platinum conjugate (complex **112**, Figure 35) as a positron and Auger electron emitting (AE) agent for the targeting of G4 DNA structures [164].

Cytotoxicity studies were performed for the non-radioactive congener of **112**, obtained with ^nat^Cu, using the HCT116 colon cancer cell line and the GM05757 normal fibroblast cell line. The non-radioactive conjugate showed a selective cytotoxicity in the tested cancer cells, being, however, less cytotoxic than cisplatin in the same cell lines. The ^64^Cu-conjugate complex was used to quantify the cellular internalization based on the measurement of the radioactivity accumulated in the cells. In agreement with the cytotoxicity results obtained for the ^nat^Cu-NOTA terpyridine platinum, the ^64^Cu congener showed significantly higher percentages of internalization in the HCT116 cancer cells as compared to the GM05757 normal cells [164]. No further studies were reported for the ^64^Cu-NOTA terpyridine platinum complex, namely the assessment of antitumor effects in cellular or animal models.

**Figure 36:**
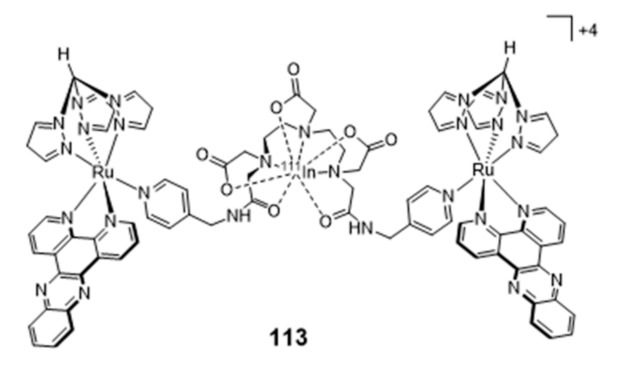
Chemical structure of ^111^In-labelled bis-ruthenium(II) dipyridophenazine theranostic complex.

By contrast, unlike G4-binders, several research groups have studied radiolabeled classical DNA intercalators aiming to deliver Auger electrons to short (sub)nanomolar distances to DNA and induce lethal DNA damage [76,165,166,167,168,169,170,171]. In particular, these studies included complexes with Auger-electron emitting radiometals, such as ^99m^Tc and ^111^In. For instance, Vallis and co-workers reported recently the synthesis of the radioactive construct [^111^In]In-bisRu(dppz) (**113**) (Figure 36) containing two DTPA-bridged ruthenium(II) polypyridyl units, which was evaluated as a potential DNA-targeting AE radiopharmaceutical [172].

As mentioned in Section 2.2, ruthenium(II)–polypyridyl complexes (RPCs) containing intercalating ligands, such as dppz, possess high DNA binding affinity with tunable selectivity for mismatch DNA. Mismatched DNA base pairs is a form of genetic instability that is prevalent in cancers showing mismatch repair (MMR) deficiency, namely in colorectal cancer. The [^111^In]In-bisRu(dppz) (**113**) radiocomplex, showing a higher affinity towards mismatch DNA over well-matched sequences, can target the nuclei of MMR-deficient cancer cells and exhibited a preferential radiotoxicity against this cell line when compared with MMR-deficient human colorectal cancer cells. However, SPECT imaging and biodistribution studies conducted with DLD-1 tumor-bearing mice showed that **113** accumulates primarily in the liver and bladder with a low tumor uptake. 

**Figure 37:**
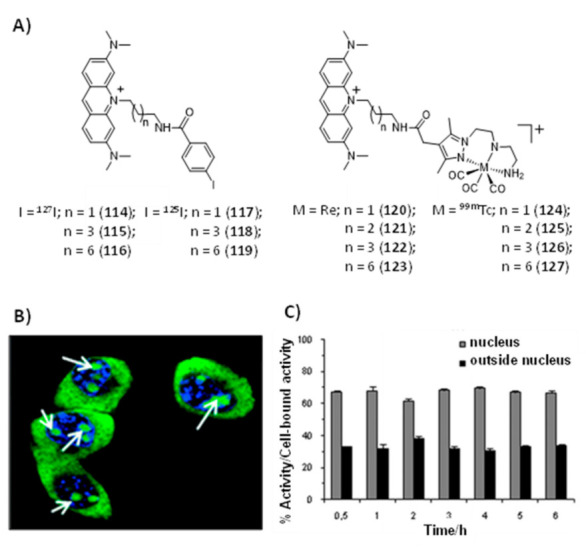
(**A**) Chemical structures of acridine orange derivatives containing natural elements (^127^I or Re) (**114**–**116**; **120**–**123**) and Auger-emitting radionuclides (^125^I or ^99m^Tc) (**117**–**119**; **124**–**127**); (**B**) Uptake of the Re complex **121** in B16F1 murine melanoma cells, as evaluated by confocal fluorescence microscopy (blue: nuclei; white arrows: nucleoli); (**C**) Nuclear internalization of the ^99m^Tc complex **125** in B16F1 murine melanoma cells, the activity values inside the nucleus and outside the nucleus are expressed as a percentage of cell-bound activity (mean ± standard deviation) [175,176].

In the field of radiolabeled DNA intercalators, Paulo and co-workers have focused on ^99m^Tc(I) tricarbonyl complexes anchored by pyrazolyl-diamine ligands functionalized with acridine orange (AO), as a classical DNA intercalator [173,174,175,176]. The first studies involved the Re(CO)_3_ complex **121** represented in Figure 37A, which contains a butylenic linker to attach the AO group to the chelator framework. It is important to notice that rhenium complexes are commonly used as surrogates of the ^99m^Tc congeners due to the physico-chemical similarities of Re and Tc complexes. Confocal microcopy studies showed that **121** internalizes and localizes in the nucleus of B16F1 murine melanoma cells (Figure 37B). 

The congener ^99m^Tc complex **125** also targets the cell nucleus exhibiting a time-dependent cellular uptake and a fast and high nuclear internalization. These encouraging results prompted an enlarged study with ^99m^Tc complexes having methylenic spacers of different length to attach the AO intercalator to the complexes, as shown in Figure 37A. In this way, the authors expected to assess how the distance of the Auger-electron emitter, ^99m^Tc, to the DNA double helix would influence the induced DNA damage and/or compromise the cell survival. These studies were complemented by the comparative evaluation of related AO derivatives labelled with ^125^I (Figure 37A), which is considered as a kind of reference Auger-electron emitting radionuclide [76]. 

As mentioned above, Paulo et al. performed a detailed and multidisciplinary investigation of the radiation-induced effects of ^99m^Tc-complexes and structurally related ^125^I-labelled derivatives, aiming to assess the influence of the distance to DNA and nature of the radionuclide on the DNA damage. This research study included the spectroscopic characterization of DNA interaction, assessment of DNA damage in vitro and in living cells, cellular and nuclear internalization in tumor cells and computational studies [176,177]. Docking simulations revealed that the compounds **118** (I = ^125^I, n = 3, Figure 37A) and **124** (M = ^99m^Tc, n = 1, Figure 37A) place the corresponding radionuclide at relatively similar distances to the axis of the DNA double helix (10.49 and 10.80 Å, respectively). This finding rendered these two compounds particularly interesting to compare the DNA damage induced by the Auger emitters ^125^I and ^99m^Tc. In fact, the results obtained in vitro for compound **124** and **126**, using plasmid DNA, showed for the first time that ^99m^Tc can induce DNA damage with an efficiency that parallels that of ^125^I, when positioned at similar distances from the DNA [176]. Furthermore, the evaluation of the ^99m^Tc complexes **120** and **122**, both in plasmid DNA and in human prostate PC3 cancer cells showed that **120** leads to more pronounced radiation-induced biological effects when compared with **122**. According to the docking simulations, the ^99m^Tc-DNA distance for complexes **120** and **122**, when intercalated into the DNA, is 10.80 Å and 12.92 Å, respectively. Therefore, the results obtained for **120** and **122** indicated that there is a marked dependence of the biological effectiveness of ^99m^Tc Auger electrons on the ^99m^Tc-DNA distance, even for a relatively small increase of such distance, as expected for short path length radiation.

Interestingly, in a collaborative research work between António Paulo and Carla Cruz groups, it was verified that the iodinated AO derivatives **114**–**116**, synthesized with non-radioactive iodine (^127^I) and presented in Figure 37A, act as potent binders of different G4 structures, namely those present in oncogenes and in human papillomavirus (HPV) genome (e.g., KRAS-22RT, c-myc, 22AG-h, 22AG-a, FHPV32T, FHPV52T or FHPV58T) [178,179]. In particular, compound **116** (designated as **C8** by the authors) showed the best affinity towards G4-DNA and exhibited a moderate cytotoxicity in different human tumor cells, as well as significant antiviral effect in HPV18-infected organotypic raft cultures. Profiting from the G4-binding ability of **C8**, its delivery to human tumor cells (e.g., human prostate PC3 cancer cells and HeLa cervical cancer cells) was studied based on a supramolecular strategy and using different G4-forming DNA and RNA aptamers recognizing the nucleolin (NCL) that is overexpressed in the target tumor cells [180,181,182,183]. In general, this supramolecular strategy improved the cancer selectivity of **C8** without compromising its cytotoxic activity. 

**Figure 38:**
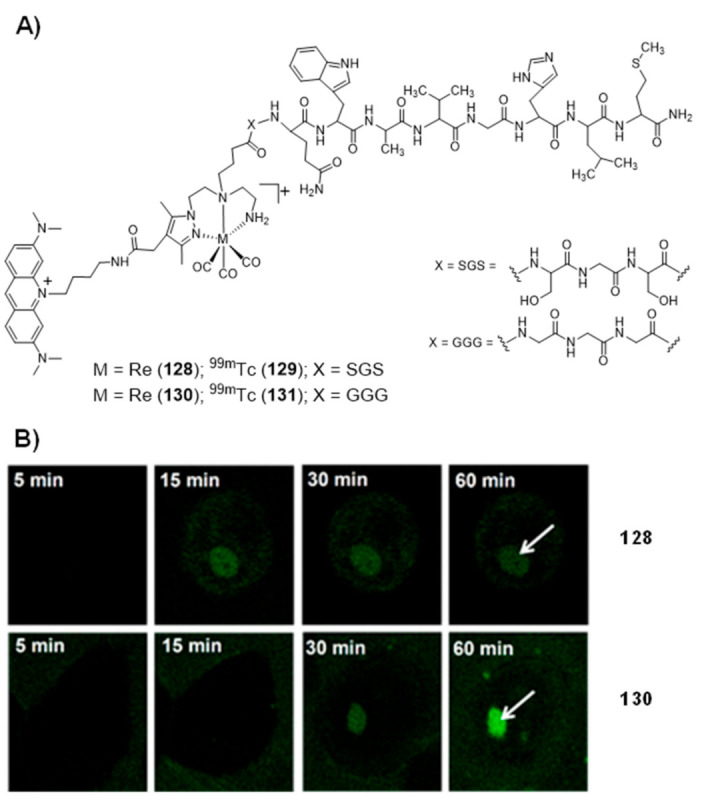
(**A**) Chemical structure of dual-targeted Re(I)/^99m^Tc(I) complexes carrying AO derivatives and BBN derivatives; (**B**) Fluorescence confocal microscopy images of live PC3 cells incubated with the Re complexes **128** and **130** ([Re] = 1.5 × 10^−5^ M). Adapted with permission from ref. [174] published by Springer Nature.

To provide AO derivatives with specificity towards cancer cells, Paulo et al. have studied a covalent bioconjugation approach AO-containing Re(I)/^99m^Tc(I) complexes carrying a bombesin sequence (BBN [7,8,9,10,11,12,13,14]) for the targeting of the gastrin releasing peptide receptor (GRPr) that is overexpressed in a variety of cancer cells (Figure 38). The Re complex **128** and the congener ^99m^Tc complex (**129**) presented a specific cell targeting with a pronounced nuclear internalization in prostate cancer PC3 cells. These results showed that multifunctional complexes can transport a DNA intercalator and an Auger-emitting radiometal, in a cell-specific way, to the nucleus of tumoral cells (Figure 38B) [174]. 

Most relevantly, the presence of a triglycine (Gly-Gly-Gly) linker between the BBN [7,8,9,10,11,12,13,14] sequence and the pyrazolyl-diamine chelator had a determinant influence on the nuclear internalization of the (radio)conjugates **129** and **131**, which was almost negligible when a Ser-Gly-Ser linker was used instead of Gly-Gly-Gly. Most probably, Gly-Gly-Gly acts as a cathepsin B cleavable linker that leads to the release of an AO-containing Re(I)/^99m^Tc(I) fragment inside the cells with better ability to reach the cell nucleus, as corroborated recently by in vitro enzymatic assays performed by the same authors with Re(I) tricarbonyl complexes [184]. The success of this dual-targeting strategy encourages the application of similar approaches to deliver classical DNA intercalators or G4-binders to the nucleus of tumor cells, aiming to achieve more selective and efficient anticancer therapies.

## 4. Concluding Remarks

Herein, we have comprehensively reviewed the most significant progresses on the design and preclinical studies of metal-based G4-binders, with a particular emphasis on fluorescent complexes that are more prone to be explored within theranostic approaches of cancer. Taking advantage on the structural diversity offered by metal complexes and their favorable fluorescent properties, the intense research developed in the past few years afforded many metal-based compounds with affinity towards G4-DNA structures and with selectivity over duplex DNA. Most of these compounds were tested in cellular models of cancer, being reported for some of them promising results in terms of their cytotoxicity profile and elucidation of mechanisms of action.

Despite these progresses, the examples of metal-based G4-binders that have been studied in animal models of cancer are rare and, to the best of our knowledge, none of them underwent clinical trials. In a way, this reflect the difficulties faced in general by metal-based compounds to be involved in biomedical translational research, from the bench to the bedside. It is our vision, that the study of radioactive counterparts might foster the translational potential of this class of compounds by facilitating biodistribution, pharmacokinetic and in vivo stability studies in cancer animal models. For this purpose, a variety of medical radiometals is available, including radioisotopes of copper and platinum that are among the most successful d- transition metals used to design complexes for selective binding to G4s. We are also convinced that it is necessary to pursue with the investigation of drug delivery tools suitable to selectively deliver metal-based G4-binders to the target cells and tissues in order to foster their potential clinical applications, either as anticancer or antiviral agents.

## Data Availability

All data is available in manuscript.
